# Recent Progress in the Development of Composite Membranes Based on Polybenzimidazole for High Temperature Proton Exchange Membrane (PEM) Fuel Cell Applications

**DOI:** 10.3390/polym12091861

**Published:** 2020-08-19

**Authors:** Jorge Escorihuela, Jessica Olvera-Mancilla, Larissa Alexandrova, L. Felipe del Castillo, Vicente Compañ

**Affiliations:** 1 Departamento de Química Orgánica, Universitat de València, Av. Vicent Andrés Estellés s/n, Burjassot, 46100 Valencia, Spain; 2 Departamento de Polímeros, Instituto de Investigaciones en Materiales, Universidad Nacional Autónoma de México (UNAM), Ciudad Universitaria, Coyoacán, Ciudad de México 04510, Mexico; olverajessica@iim.unam.mx (J.O.-M.); laz@unam.mx (L.A.); lfelipe@unam.mx (L.F.d.C.); 3 Departamento de Termodinámica Aplicada (ETSII), Universitat Politècnica de València, Camino de Vera. s/n, 46022 Valencia, Spain

**Keywords:** fuel cells, proton exchange membrane, polymer, polybenzimidazole, composite membranes, conductivity, carbon nanotubes, graphene oxide, ionic liquids, metal organic frameworks

## Abstract

The rapid increasing of the population in combination with the emergence of new energy-consuming technologies has risen worldwide total energy consumption towards unprecedent values. Furthermore, fossil fuel reserves are running out very quickly and the polluting greenhouse gases emitted during their utilization need to be reduced. In this scenario, a few alternative energy sources have been proposed and, among these, proton exchange membrane (PEM) fuel cells are promising. Recently, polybenzimidazole-based polymers, featuring high chemical and thermal stability, in combination with fillers that can regulate the proton mobility, have attracted tremendous attention for their roles as PEMs in fuel cells. Recent advances in composite membranes based on polybenzimidazole (PBI) for high temperature PEM fuel cell applications are summarized and highlighted in this review. In addition, the challenges, future trends, and prospects of composite membranes based on PBI for solid electrolytes are also discussed.

## 1. Introduction

Proton conductivity has received intense attention owing to its application in chemical sensors, electrochemical devices, and power generation [1,2]. Proton conducting membranes form an important part in fuel cells (FCs), batteries, and supercapacitors [3]. Fuel cells based on proton exchange membranes (PEMs) are one of the most promising alternative energy sources because of their high efficiency, high power density, low emissions, and energy supply [4,5]. These alternatives provide the possibility of receiving energy from hydrogen, synthetic, or bio-synthetic fuels and can operate with greater efficiency and environmental sustainability compared with thermal motors [6,7]. Fuel cells can be used for a wide variety of technological devices such as vehicles, mobile phones, portable electronics, and power generators [8,9,10]; they are generally classified by the kind of electrolyte they use. Common classifications include alkaline fuel cells [11], direct methanol fuel cells [12], polymer electrolyte membrane fuel cells [13], phosphoric acid fuel cells [14], molten carbonate fuel cells [15], solid oxide fuel cells [16], and reversible fuel cells (also called unitized regenerative fuel cells) [17].

**Figure 1:**
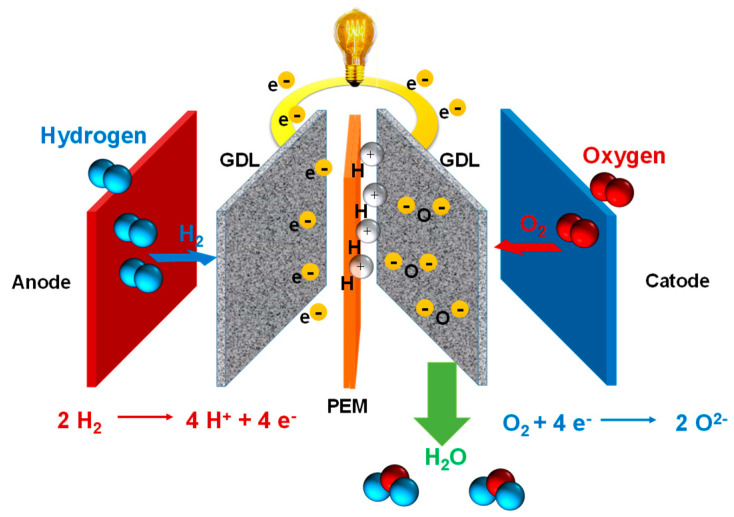
Schematic flow of PEMFC.

**Figure 2:**
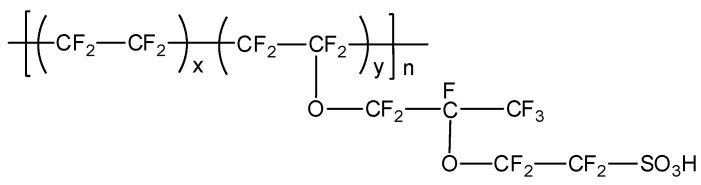
Chemical structure of Nafion^®^.

In a typical proton exchange membrane fuel cell (PEMFC), the polymer electrolyte membrane is responsible for proton conductivity, which allows the transport of protons from the anode to the cathode, constituting the essential component of the electrochemical device [18]. PEMFCs constitute a promising alternative to fossil fuels as they generate water as a byproduct and use only hydrogen and oxygen as reactants (Figure 1). According to their range of operating temperatures, PEMFCs can be classified into three main categories: (a) low temperature PEMFCs (LT-PEMFCs), which operate around 50–80 °C [19]; (b) intermediate temperature (IT-PEMFCs), which operate in the 80–120 °C range [20,21,22]; and (c) high temperature (HT-PEMFCs), which operate from 140 °C up to 200 °C [23,24], generally under anhydrous conditions. Among the numerous types of PEMs, membranes based on perfluorosulfonic acid polymers, such as Nafion^®^ (Figure 2), have wide acceptance as they have been demonstrated to possess good conductivity as well as chemical and mechanical properties, have been used at temperatures below 90 °C, and have endured conditions of high relative humidity [25].

Nafion^®^, developed by DuPont in the late 1960s, still remains as the state-of-the-art membrane for low temperature PEMFCs (LT-PEMFCs). The main drawbacks of Nafion^®^ membranes are mainly their costly manufacturing process, the destruction of the polymer structure at higher temperatures, and the strong decrease in proton conductivity at temperatures above 90 °C when low hydration conditions are reached [26,27,28,29,30]. Consequently, Nafion^®^-based membranes are limited to operate as LT-PEMFCs. These practical limitations have promoted the development of membranes that may be applied in HT-PEMFCs operating temperatures in the range of 140–200 °C and, hence, in the absence of water [31,32,33,34,35].

Among the benefits of working at high temperatures, it is worth mentioning a decrease in the catalyst contamination by CO and CO_2_ poisoning, as the kinetics in the electrodes are faster and have a simpler thermal and water handling, low dependency on cooling systems, a high amount of reusable heat energy, as well as a lower cost of the membrane-electrode assemblies (MEAs) in comparison with LT-PEMFCs based on Nafion^®^ [36,37,38]. The high CO tolerance of the anode catalyst makes it possible for an FC to use hydrogen directly from a simple methanol reformer, thus the selective oxidant and/or CO separator device can be removed from the processing system. Consequently, the size of an FC is importantly reduced, improving its performance, responsiveness, and reliability, which ultimately allows to bring down system maintenance and operating costs [39]. In order to optimize the performance of FCs, a lot of work has been done in the development of HT-PEMs, particularly on those based on polybenzimidazoles (PBIs), which have emerged as promising candidates to operate at high temperatures [40,41,42,43,44].

**Figure 3:**
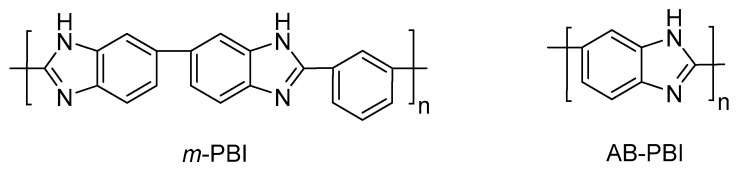
Chemical structures *m*-PBI and AB-PBI.

PBIs are aromatic linear heterocyclic macromolecules that belong to the class of high performance polymers; they are highly resistant to acids and bases; have high glass transition temperatures (425–436 °C); and possess excellent thermal and mechanical stability, low flammability, and high energy radiation resistance. Aromatic polybenzimidazoles were firstly synthesized by H. Vogel and C. S. Marvel in 1961 [45], and as a consequence of their exceptional thermal and oxidative stability, they were used on aerospace and defense applications by NASA and the Air Force Materials Laboratory (AFML), respectively. The most widely studied polybenzimidazole is one commercialized by the corporation Celanese, poly [2,2′-(*m*-phenylene)-5,5′-bibenzimidazole], or *m*-PBI (also known as simply PBI). This polymer can be synthesized by a polycondensation reaction using 3,3′-diaminobenzidine (DAB) and isophthalic acid (IPA) [46,47]. Another available polybenzimidazole is poly (2,5-benzimidazole) (or AB-PBI), which was also carefully studied as membrane for HT–PEMFCs. The AB-PBI is prepared by condensation of 3,4-diaminobenzoic acid (DABA) [48]. The chemical structures of both *m*-PBI and AB-PBI are shown in Figure 3.

However, to achieve the high proton conductivity levels required for HT-PEMFCs, these simple PBIs need to be doped with acid because their intrinsic conductivity is very low (around 10^−12^ S/cm). In particular, phosphoric acid (PA)-doped PBI membranes demonstrated good thermal and mechanical stability, low gas permeability, and conductivity values between 0.07 and 0.2 S/cm at approximately 200 °C without any additional humidification [49,50]. As the ionic conductivity of PA is low at low temperatures, PBI embedded in PA operating temperature range is about 150–250 °C. The hydrogen oxidized at the anode splits into protons and electrons. The electrons pass through the external electrical circuit, whereas the protons are transferred through the electrolyte. On the cathode side, the redox reaction between positive hydrogen ions, electrons, and oxygen gas results in water formation [51]. The main advantage of phosphoric fuel cells is their capacity to generate and separate electricity and useful heat at the same time. However, the use of this acid-doping method presents various inconveniences, limiting the applications; particularly, the pyrolysis of PA above 190 °C [50], the migration of PA resulting in loss of transition metal catalyst and reducing proton conductivities, acid leaching problems, and reduced mechanical properties under HT-PEMFCs’ operation conditions.

Another efficient method to increase conductivity is the introduction of the covalently bound sulfonic and/or phosphoric groups into the backbone chains, but multi-step complex syntheses are generally needed for the formation of such PBIs [52,53] Additionally, the aggressive polymerization conditions may affect the side-groups, resulting in a crosslinked polymer gel instead of a linear structure. Consequently, the possible structural variations in the polymers are limited and PBIs containing functional groups in their main chain are scarce. PBIs obtained by changing one of the monomers allow for the modification of the physico-chemical properties (e.g., basicity), simply by playing with the number of the nitrogen atoms in the monomer and their distribution along the polymer [54]; in this manner, acid leaching can be minimized [55].

**Figure 4:**
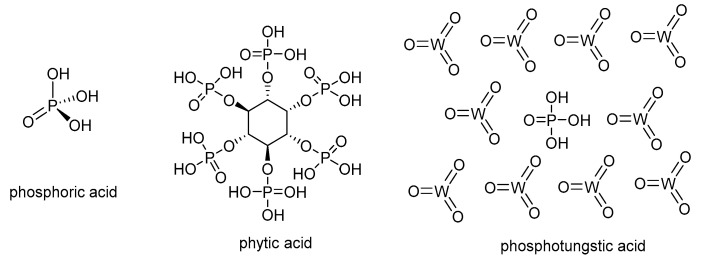
Chemical structures of phosphoric acid, phytic acid, and phosphotungstic acid.

The doping strategies for PBI-type membranes consist of an incorporation of significant amounts of phosphoric acid to the polymer matrix to achieve sufficient proton conductivity. The acid doping can be performed in various ways. The simplest and most efficient method consists of directly immersing the PBI-type membrane sheet into hot phosphoric acid. The doping level of the membrane depends on the immersion time and temperature. As an example, the AB-PBI based-membrane doped at 120 °C for 24 h can absorb phosphoric acid up to 2.5 times its own weight, which corresponds to the chemical formula AB-PBI·5H_3_PO_4_. On the other hand, the thickness practically doubles during the doping process. For example, the thickness changed from 50 μm for a pristine PBI membrane to almost 100 μm when it was fully doped [56]. The interaction between acid and the polymer matrix occurs via the N-imidazole sites. The basic N-sites of PBI act as proton acceptors like in a standard acid–base reaction, creating the ion pairs in this process as shown in Figure 4. Therefore, the polymer bearing more basic N-sites forms stronger bonds with acids.

The conductivity depends not only on the doping level, but also on the acid distribution within the membrane. In this sense, the membranes should ensure that the acid percolates throughout the polymer network and interacts with nearly every basic N-site of the polymer matrix. Finally, the conductivity of composite membranes based on PBIs is also affected by the dehydration reaction of phosphoric acid. It was noticed that, under anhydrous conditions, the conductivity of phosphoric acid decreases for temperatures above 140 °C owing to a condensation reaction of PA affording pyrophosphoric acid (H_4_P_2_O_7_) and water. Therefore, at temperatures between 140 and 180 °C, the cell resistance of MEA based on PBIs under open circuit conditions is significantly higher than that with an electrical load and, additionally, it allows to produce water through the fuel cell reaction. The water formed permits the rehydration of the membrane in the MEA and results in a better conducting phosphoric acid [57].

Despite the wide use of PA as acid doping for PBI-based membranes, other acids have been used in the past years to overcome the drawbacks associated with the use of PA, mainly acid leaching and corrosion, which can seriously affect the long-term stability of fuel cells. Among other doping acids containing phosphonate groups, phytic acid (myoinositol hexakisphosphate) is considered a sustainable alternative to phosphoric acid (Figure 4). Phytic acid is a phosphorus-containing organic acid that can be found in plants, especially in seeds and fibers. This acid has been used as a doping agent in combination with metal organic frameworks (MOFs) for polymer electrolyte membranes based on Nafion^®^, reaching high proton conductivities [58,59]. Because of its molecular size, this natural acid can be encapsulated into cavities, reducing the leaching from the membrane. Another type of alternative acid is heteropolyacids, which are a particular class of acids made up of a combination of hydrogen and oxygen with metals and non-metals. Among this family of heteropolyacids, phosphotungstic acid (HPW), with molecular formula H_3_P_4_W_12_O_40_, has been efficiently applied as a proton carrier in proton exchange membrane fuel cells [60,61]. Some representative literature on heteropolyacid doped polymer as PEM should be consulted [62,63]. In a recent work, we have evaluated the long-term stability of PBI membranes doped with different concentrations of the widely used PA, and an important acid leaching and subsequent conductivity drop was observed [64].

## 2. Proton Conduction and Transport Mechanism

As has been previously commented, the proton conductivity of acid-doped PBI membranes is strongly dependent on the physico-chemical parameters such as temperature, relative humidity (RH), as well as the acid doping level measured in molar concentration of the doping solution [65,66]. Such conductivity is generally accepted to occur by two different mechanisms [67]: the Grotthuss mechanism (also known as hopping mechanism) and the vehicle mechanism. The prevalence of either the hopping or vehicle mechanism depends on the hydration level of the membrane. On the other hand, the mechanism of proton transport in composite and hybrid membranes is much more complex as it involves both the surface and chemical properties of the inorganic and organic phases present in the composite membrane.

**Figure 5:**
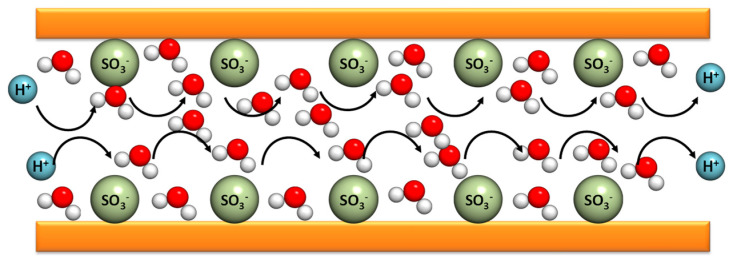
Schematic representation of the Grotthuss mechanism in a polymeric membrane.

The Grotthuss mechanism is characterized by the protons’ jumping from one site to another along a hydrogen-bonding (HB) chain. In the case of PBI composite membranes, the protons are transferred from the nitrogen benzimidazole sites (N–H) to the phosphoric acid anions inside of the polymer matrix. This contribution is relevant for n = [H_3_PO_4_]/[PBI] < 2. When *n* > 2, there is a possibility of a proton hopping along the phosphoric acid anions. This mechanism is relevant in the case of non-doped PBI. The original idea was proposed by Theodore von Grotthuss in 1806 to explain a mechanism for proton (H^+^) transport between water molecules [68]. As shown in Figure 5, the protons jump from a protonic species such as H_3_O^+^ to another protonic species in the membrane [69].

**Figure 6:**
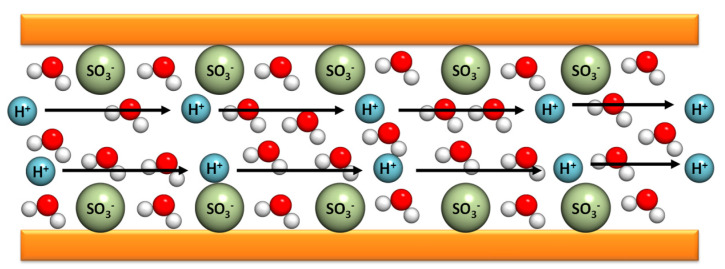
Schematic representation of the vehicular mechanism in a polymeric membrane.

The second mechanism, namely the vehicle mechanism, was proposed in 1982 by Kreuer, Rabenau, and Weppner [70] and rationalizes that the motion of protons is assisted by carrying molecules that, in general, are associated to the doping acidic groups. This last mechanism accounts for the major contribution for the conductivity increment of the polymeric membranes. Finally, a combination of the two described mechanisms can be observed in the case of diffusion of protons as part of polymeric structures involving molecules of water such as H_3_O^+^, H_5_O_2_^+^ (Zundel cation), H_9_O_4_^+^ (Eigen cation), or some other species, such as NH_4_^+^, which may also be present in the polyelectrolyte, in combination with the diffusion of vehicles as uncharged molecules. As illustrated in Figure 6, the protons attach themselves to a vehicle site such as water, which is diffused through the medium, and thus carry the protons along.

When the protons are moving through a hopping mechanism, the conductivity of the acid-doped PBI is governed by an activation mechanism that obeys the Arrhenius law [71], where the relation between the conductivity and temperature is governed by the expression.

(1) $$\sigma =A\;\exp\left(-\frac{E_{a}}{\mathrm{RT}}\right),$$

where σ (S/cm) is the proton conductivity at a certain RH, A is a pre-factor, E_a_ (kJ/mol) is the activation energy at a certain RH, R (8.314 J/mol K) is the gas constant, and T (K) is the absolute temperature.

The values of E_a_ for *n* > 2 are in the range 15–25 kJ/mol, very near to that of concentrated H_3_PO_4_ aqueous solution. In this situation, PBI-based membranes doped with acidic groups show high proton conductivity if properly doped with strong acids such as H_3_PO_4_ depending on the amount of PA absorbed into the porous of its matrix. In fact, PBI is a basic polymer, which dissociates PA releasing protons, as sketched by the following reaction: H_3_PO_4_ + PBI → H_2_PO_4_^−^ + PBI^−^H^+^, where the equilibrium constant is about K = 1.17 × 10^3^ and H^+^ ions can migrate through the polymer backbone by means of hydrogen bonds, in this case, assisted by phosphate anions by means of the Grotthuss mechanism, as we previously mentioned.

In some cases, such as PA-doped composite membranes of PBI containing ionic liquids, the dependence of the conductivity versus temperature presents a different behavior than the typical Arrhenius behavior and the activation energy associated with the conductivity mechanism is not constant for the entire the range of temperatures. In such a situation, the conductivity of the membranes exhibits a Vogel–Tammann–Fulcher (VTF) conduction behavior, where proton hopping is coupled with the segmental motion of the polymer chains and the activation mechanism is given according to the equation.

(2) $$\ln\sigma_{dc}=\ln\sigma_{\infty }-\frac{B}{T-T_{0}},$$

where *B* is the fitting parameter related with the curvature of the experimental data plot; *T*_0_ is the Vogel temperature, which is considered as the one at which the relaxation time would diverge; and *σ*_∞_ is the pre-factor related with the conductivity limit at high temperatures. 

**Figure 7:**
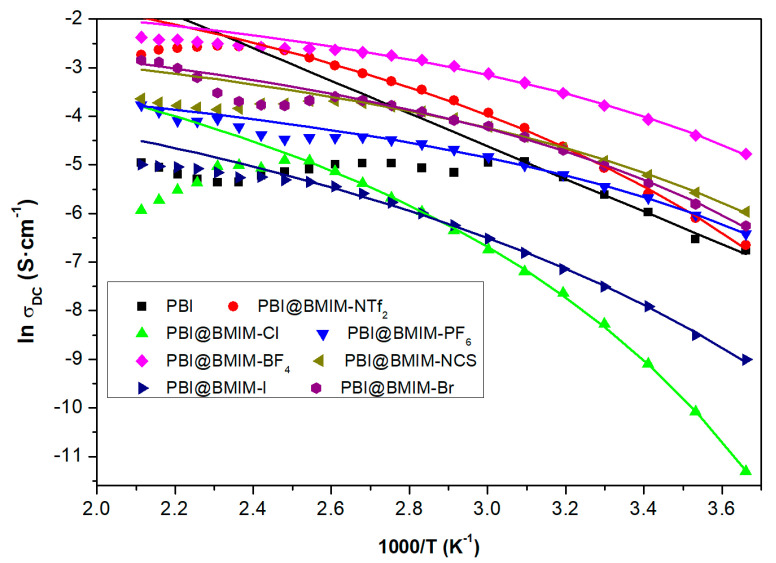
Variation of the ln σ_dc_ as a function of the reciprocal of the temperature for phosphoric acid doped PBI composite membranes containing 5 wt.% of 1-butyl-3-methylimidazolium (BMIM)-X (X: [NTf_2_]^−^, [Cl]^−^, [BF_4_]^−^, [I]^−^, [PF_6_]^−^, [NCS]^−^ and [Br]^−^). For comparison, the conductivity dependence with temperature for PBI pristine membrane doped with phosphoric acid is also given.

Figure 7 displays the results found for the conductivity of some composite membranes of PBI filled with ionic liquids (ILs), where a behavior different from linear is observed depending on the IL type. The activation energy associated with this kind of composite membrane is much higher for lower temperatures than the one associated with higher temperatures, in agreement with the experimental results. The values for the activation energy obtained from the fit of Equation (2) to the experimental data plotted in Figure 7 follow the trend [Cl]^−^ (6.33 kJ/mol) > [I]^−^ (5.80 kJ/mol) > [NTf_2_]^−^ (5.35 kJ/mol) > [Br]^−^ (3.04 kJ/mol) > [NCS]^−^ (2.91 kJ/mol) > [BF_4_]^−^ (2.53 kJ/mol) ≈ [PF_6_]^−^ (2.51 kJ/mol). A close inspection of Figure 7 reveals a change in the slopes. For PBI@BMIM-NTf2 and PBI@BMIM-Cl, a negative slope is observed at high temperatures, in contrast with a slightly positive slope for PBI@BMIM-NCS and PBI@BMIM-BF4. This behavior can be associated with the variation in Debye’s length, which is related to the effective dissociation energy and the measured dielectric permittivity in the absence of electrode polarization as well as of orientational polarization of dipolar ions, as previously reported [72,73].

There are many examples of PBI composite membranes with values of proton conductivity and its corresponding activation energy. As an example, Wang and et al. [74] prepared an insoluble sulfonated polyphosphazene (SPOP) with 117% degree of sulfonation; the SPOP was used as the proton conductor in the PBI as HT-PEM, and the polyfunctional triglycidyl isocyanurate (TGIC) was used as a covalent cross-linking agent to inhibit swelling at low degree of cross linking of the PBI-TGIC/SPOP composite membranes obtained. The proton conduction activation energy was calculated at a specific set of RH and is summarized in Table 1. The conductivity of PBI-TGIC(5%)/SPOP(50%) at 100% RH, 50% RH, and 0% RH is 0.143, 0.076, and 0.044 S/cm at 180 °C, respectively. The authors described that the proton conduction activation energy increased with the increasing degree of crosslinking. This inhibited the water absorption of the composite membrane and hindered the passage in the membrane of the proton conduction through the vehicle mechanism. On the other hand, the increase of the doping amount of SPOP reduced the proton activation energy, because it promoted water absorption of the membrane and improved the connectivity of the hydrophilic regions in the membrane. When the RH% was lowered, the efficiency of the vehicle mechanism for conducting protons was reduced, thereby causing a decrease in proton conductivity and an increase in proton conduction activation energy. At 0% RH, the proton conduction mechanism of the PBI-TGIC/SPOP composite membrane is the hopping mechanism.

Recently, Rajabi et al. prepared composite membranes by introducing melamine-based dendrimer amines (MDA) with mesoporous silica (SBA-15), and 1,3-di(3-methylimidazolium) propane dibromide dicationic ionic liquid (pr(mim)_2_Br_2_) (DIL), the membranes were doped with PA and were used to improve the proton conductivity [75]. The APBI-DIL_4.5_-MDA_1.5_ composite membranes exhibit a proton conductivity of 0.22 S/cm at 180 °C under dry conditions. With the increasing temperature, the interaction between polymer chains decreases, leading to more mobility of proton carriers. The high mobility of protons and their access to the active sites can also have a positive effect on the E_a_ required for proton conduction through the Grotthuss and vehicular mechanisms in the membranes. According to the results displayed in Table 2, APBI-DIL_4.5_-MDA_1.5_ composite membranes require a lower E_a_ to conduct the proton compared with the other membranes.

Compañ’s working group [64] presents a systematic study of the physico-chemical properties and proton conductivity of PBI membranes doped with PA and other acids such as phytic acid and phosphotungstic acid (HPW). To further study the proton conduction mechanism of the membranes, the Arrhenius plots of the membranes and their proton conduction activation energy values (E_a_) were analyzed; the results are presented in Table 3. Proton conductivity increases for all membranes from 20 to 180 °C, following typical Arrhenius behavior. The obtained values followed the trend: E_a_ (PBI–PA 14 M) < (PBI–PA 1 M) ≈ (PBI–phytic) < (PBI–PA 0.1 M) < (PBI–HPW). These results indicate that proton mobilities increased with the amount of PA, and then PA formed channels in the organic phase of porous PBI. Similar activation energies were obtained for PBI–PA 1 M, PBI–PA 0.1 M, and PBI–phytic acid membranes, but PBI–PA 14 M displayed a lower value as the PA concentration was much higher and, therefore, the proton transport was more favored. According to these results, the Grotthuss mechanism dominates the proton transport in acid doped PBI membranes.

In a recent publication of the group of Compañ, SiO_2_ nanofiber mats were used as fillers in the preparation of composite PBI membranes for high temperature PEMFC applications [76]. In this work, SiO_2_ nanofibers were fabricated through an electrospinning process and later functionalized using silane chemistry to introduce different polar groups, namely, −OH (neutral), −SO_3_H (acidic), and −NH_2_ (basic). The modified nanofiber mats were embedded in PBI to fabricate mixed matrix membranes. Proton conduction measurements show that PBI composite membranes containing nanofiber mats with basic groups showed higher proton conductivities. The E_a_ values of the composite membranes under wet conditions were lower than that for the pure PBI membrane, which is associated with an enhanced proton mobility, as the nanofiber acts as a carrier-bridge for protons and, consequently, the process demands less energy. On the one hand, the Grotthuss mechanism explains the conductivity by means of the interaction of protons through the jump between a hydrogen bond network of N−H groups, both from the PBI and from the functionalized groups of the nanofiber mats. On the other hand, protons can move via the vehicle mechanism through the hydroxyl, amine, or sulfonic groups from the different nanofiber functionalization and imidazole groups present in the PBI, which may interact with water molecules, promoting the proton conductivity. Additionally, activation energies under dry conditions improve proton conduction, which is mainly owing to a vehicular mechanism, as inferred from the calculated activation energies, with values higher than 55 kJ/mol (see Table 4).

In 2018, our research group described the preparation and characterization of composite PBI membranes containing zeolitic imidazolate framework (ZIF-8) and (ZIF-67) [77]. The calculated E_a_ associated with the proton transport in the PBI−ZIF-8 and PBI−ZIF-67 and PBI−ZIF−mix are shown in Table 5. Considering that the E_a_ for the PBI-ZIF membranes is lower than that for pure PBI membrane, it is suggested that the ZIFs clearly favor the conductivity of the membrane at moderate and high temperatures.

**Figure 8:**
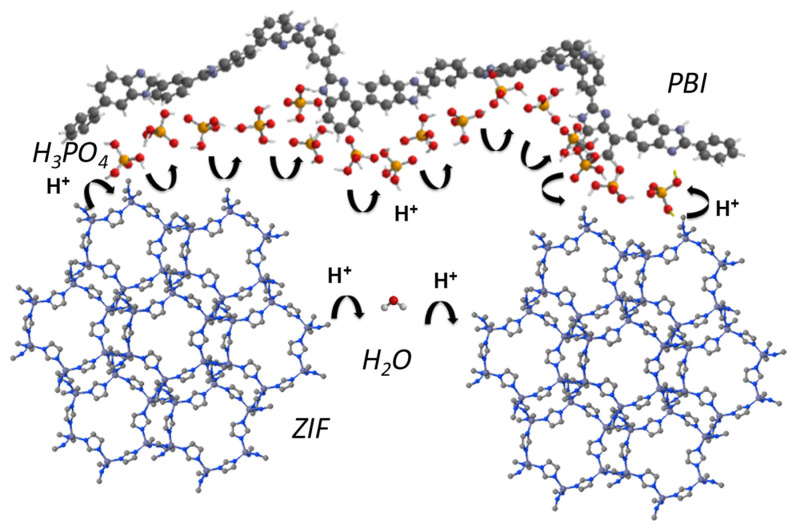
Schematic illustration of the possible mechanism for proton transfer in zeolitic imidazolate framework (ZIF)-containing PBI composite membranes. C, N, O, and P atoms are represented as grey, blue, red, and orange, respectively. H atoms are omitted from the ZIF structure for clarity [77].

According to the calculated activation energies, the proton conductivity could be rationalized by means of the Grotthuss mechanism by proton hopping through a network of hydrogen bonds along the polymeric matrix involving polybenzimidazole polymeric chains, phosphoric acid networks, and imidazolate rings from the ZIF (Figure 8).

Abouzari-Loft and co-workers used the 2,6-pyridine functionalized polybenzimidazole (Py-PBI) as substrate for hosting PA moiety. Phosphonated graphene oxide (PGO) was added to Py-PBI substrate at different levels prior to acid doping [78]. They found that the conductivity is more stable in the Py-PBI-PGO membranes. The proton conductivity of the membranes showed a temperature dependence, that is, an Arrhenius-type of behavior in the whole range of temperatures. The activation energy for the Py-PBI based membrane was lower for the composite membranes with 1.0% and 1.5% PGO contents, as shown in Table 6. The authors proposed that such a reduction in the E_a_ for the PGO containing membranes may indicate the synergic effect of the phosphonated PGO filler in boosting the membrane proton conductivity. The presence of PGO with an exfoliated structure may have disrupted the crystalline structure of PyPBI, which results in more available and stronger sites for PA trapping and provided additional diffusion pathways for proton hopping across the membrane via the Grotthuss mechanism.

## 3. Influence of the PBI Structure and Synthetic Methods on Its Conductivity

As mentioned above, PBIs, owing to their excellent mechanical properties and high chemical resistance, are considered as the most important polymers for the fabrication of membranes to work at high temperatures in the construction of stacks for HT-PEMFCs. The PBI membranes are generally prepared by casting from polymer solutions and then doped with acid, although a direct method for casting the PBI together with acid has also been developed [79,80].

The structure of PBI has an important effect on many of its properties such as solubility, stability, and proton conductivity. PBIs have a poor solubility owing to their rigid structure. Another important factor affecting the solubility is the molecular weight of the polymer. In this regard, PBIs with molecular weights ranging from 23 to 37 kDa have relatively good solubility, but polymers with lower molecular weights are not strong enough to ensure fuel cell requirements. However, polymers with higher molecular weights are found to be poorly soluble in polar organic solvents. Therefore, many approaches have been used to improve both the strength and solubility of PBI [81,82].

**Figure 9:**
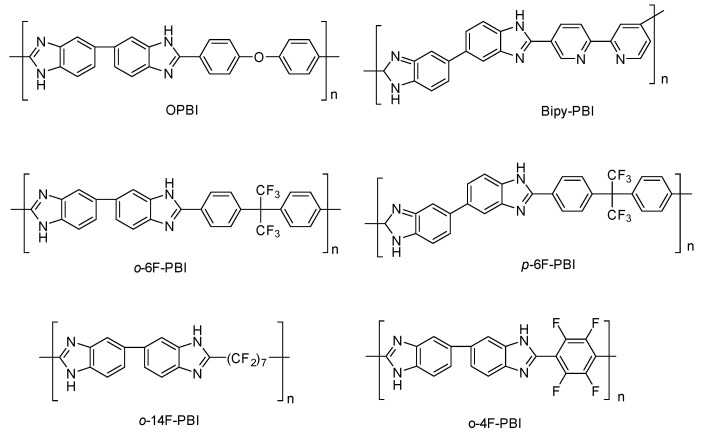
Different structures of PBIs.

**Figure 10:**
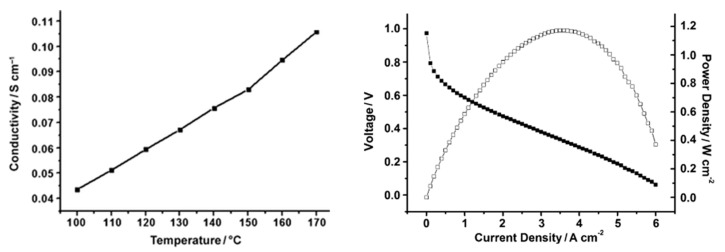
Variation of conductivity of PA doped OPBI with temperature (**left**) and fuel cell performance (▪: voltage, □: power density) of the OPBI membrane (**right**). Reproduced from [83] with permission of John Wiley and Sons.

One strategy to strengthen and make PBI more soluble consists of introducing a flexible group in the backbone, such as ether linkages, that facilitates the chain mobility, and consequently solubility, and simultaneously enhances the molecular weight of the PBI and its strength. PBIs containing ether units have been synthesized and applied as polymeric electrolytes for HT-PEMFCs. Furthermore, the introduction of ether units in the PBI has been reported an effective method of increasing the proton conductivity of the membranes. So, the synthesis of poly [2,2′-(*p*-oxydiphenylene)-5,5′-benzimidazole] (OPBI) obtained by polycondensation of 3,3′-diaminobenzidine (DAB) and 4,4′-oxybisbenzoic acid (OBBA) was reported (Figure 9), and the membrane prepared from this polymer, after PA doping, which showed values of proton conductivity up to 0.083 S/cm at 150 °C under anhydrous conditions [83]. The fabricated PBI/PA MEA was tested for 100 h without any degradation in voltage noticed, reaching maximum power and current densities of 1.17 W/cm^2^ and 6.0 A/cm^2^, respectively (Figure 10).

In 2019, Berber and Nakashima described the synthesis of bipyridine-based polybenzimidazole (Bipy-PBI) of various molecular weights and it was concluded that the molecular weight significantly affected thermal stability, mechanical properties, proton conductivity, and FC performance of the membranes [84]. The conductivity of the 141 kDa Bipy-PBI membrane reached 0.037 S/cm at 120 °C and anhydrous conditions, with a maximum power density of 0.78 W/cm^2^ and a current density at 0.5 V of 1.6 A/cm^2^.

An alternative synthesis of OPBI by microwave irradiation was also reported by Kang and coworkers [85]. The proton conductivity of the H_2_SO_4_-doped membranes prepared from this PBI showed values up to 0.190 S/cm at 160 °C and anhydrous conditions. The synthesis of porous and asymmetric OPBI without the use of any porogenic additive was also developed by Ou and coworkers; the conductivity of 0.072 S/cm at 180 °C for such OPBI membrane was reported and a peak power density of 0.4 W/cm^2^ at 160 °C under anhydrous conditions was reached in the fuel cell test [86].

The incorporation of hexafluoroisopropylidene groups in the polymeric structure has been efficiently used to improve the proton conductivity of polyimides and polyamides [87]. Consequently, such an approach was tried on PBI membranes. In this regard, Qian and Benicewicz prepared a hexafluoroisopropylidene-containing *o*-polybenzimidazole (*o*-6F-PBI) membrane with a proton conductivity of 0.09 S/cm at 180 °C after phosphoric acid doping and a fuel cell performance with a maximum power density of 0.58 W/cm^2^ 0.2 A/cm^2^ was described [88]. A year later, another hexafluoroisopropylidene-containing PBI (*m*-6F-PBI) was synthesized and the PA-doped membrane exhibited a proton conductivity of 0.02 S/cm at 180 °C, and a peak power density of 0.574 W/cm^2^ at 0.2 A/cm^2^ at 180 °C. The synthesis of other fluorinated PBIs, such as poly (2,2′-(tetrafluoro-*p*-phenylene)-5,5′-bibenzimidazole) (*o*-4F-PBI) and poly(2,2′-tetradecafluoroheptylene-5,5′-bibenzimidazole) (*o*-14F-PBI), has been described, and their PA-doped membranes reached conductivity values up to 0.03 S/cm at 150 °C. The crosslinked *m*-6F-PBI membranes using epoxide cross-linkers were prepared. The membranes showed high acid doping level, enhanced mechanical strength, and oxidation stability in comparison with the pure *m*-6F-PBI. Additionally, the proton conductivity in these cross-linked PA-doped membranes reached values up to 0.060 S/cm at 160 °C and a current density of 0.634 A/cm^2^ at 0.51 V [89].

Chemical modification of PBI by means of click chemistry reactions [90] and by the alternative and enviromental friendly metal-free click chemistry reactions [91], such as the strain-promoted azide–alkyne cycloaddition (SPAAC) [92], the thiol-ene [93,94] and thiol-yne coupling [95,96], the inverse electron−demand Diels−Alder (IEDDA) reaction [97], the strain-promoted alkyne−nitrone cycloaddition (SPANC) [98,99], and the strain-promoted oxidation-controlled cyclooctyne–1,2–quinone cycloaddition (SPOCQ) [100,101,102], can afford an interesting variety of PBI structures with potential applications in PEM for fuel cell applications. The main advantages of these reactions include their fast reaction kinetics, versatililty and regiospecificity, high product yields, and easy purification of the products. In this regard, these methodologies have been efficiently used along the past decade in the preparation of polymers [103], but their application in fuel cells remains almost unexplored.

## 4. Copolymers

**Figure 11:**
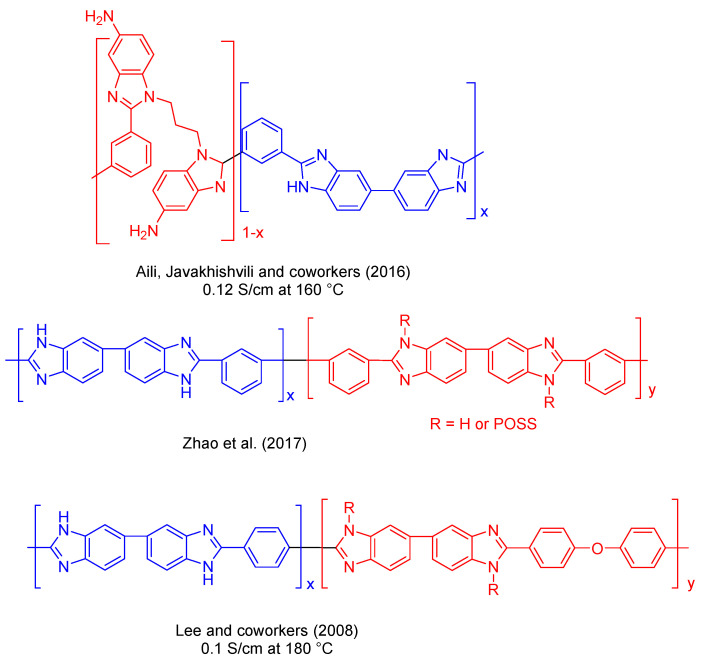
Different copolymers of PBI. POSS, polyhedral oligosilsesquioxane.

Another commonly used alternative to fabricate PBI-based polymers with desirable properties (high proton conductivity in combination with high chemical and thermal stability and adequate mechanical properties) is based on the preparation of PBI copolymers [104]. On these lines, copolymerization has attracted researchers’ interest in the last decade as it allows a fine tuning of the polymer properties by simply selecting the monomer concentration/monomer ratio in the copolymer (Figure 11) [105].

Aili, Javakhishvili, and coworkers have synthesized an amino-functional benzimidazole copolymer by condensation of isophthalic acid, 3,3′-diaminobenzidine, and new a hexamine, *N*,*N*′-bis(2,4-diaminophenyl)-1,3-diaminopropane (Figure 11). Blend membranes prepared with conventional PBI showed proton conductivities up to 0.12 S/cm at 160 °C after phosphoric acid doping [106], which was in accordance with the observed high acid uptakes. Zhao et al. synthesized a series of grafted polybenzimidazole copolymers containing polyhedral oligosilsesquioxane (POSS) pendant moieties, which are a family of well-defined cage-like molecules (Figure 11) [107]. These copolymers exhibited improved mechanical properties over pristine PBI, with a Young’s modulus of ∼5 GPa and tensile strength of 85 MPa; whereas for pristine PBI, the values were of 1.36 GPa and 71 MPa, respectively. Another copolymerization approach described by McGrath and coworkers was based on multiblock copolymers containing different block lengths. These copolymers were prepared by coupling carboxyl functional aromatic poly (arylene ethers) with ortho diamino functional PBI oligomers in different poly(arylene ether sulfone) and PBI ratios [108]. Using a similar approach, Lee and coworkers described the synthesis of poly (aryl ether benzimidazole) copolymers containing different contents of aryl ether linkages by condensation of 4,4-dicarboxydiphenyl ether (DCPE) and terephthalic acid (TA) by varying the DCPE/TA ratio (Figure 11). The authors concluded that the optimal content of aryl ether linkages was 10–30 mol %. With this approach and after PA doping, membranes displayed a proton conductivity of 0.1 S/cm at 180 °C [109].

**Figure 12:**
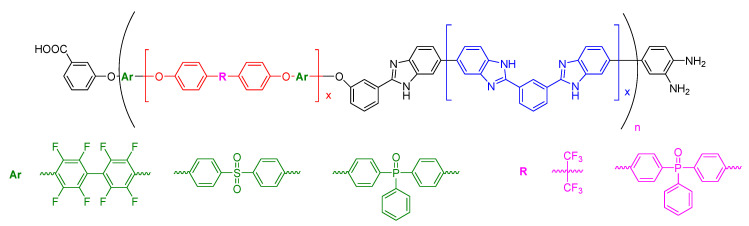
PBI-based block copolymers of phosphophilic PBI and phosphophobic non-PBI segments [115].

The group of Benicewicz has deeply explored the synthesis of new compositions to improve the chemical, thermal, and mechanical stability, as well as proton conductivity, of PBI-based membranes. In 2005, a series of *meta*/*para*-PBI random copolymer membranes were fabricated by Benicewicz and coworkers via a phosphoric acid sol–gel process. These copolymers exhibited a maximum proton conductivity of 0.26 S/cm at 180 °C with a high acid doping level, which was efficiently retained in the membrane [110]. Fuel cell tests displayed a current density of 0.2 A/cm^2^ and a voltage around 0.65 V at temperatures above 150 °C without any feed gas humidification, and were stable for more than 1000 h. In 2011, Mader and Benicewicz reported the preparation of PBI containing sulphonic acid groups by copolymerization of *p*-PBI with polyphosphoric acid (PPA), which displayed excellent proton conductivities with values close to 0.3 S/cm at 180 °C [111]. The authors used different segmented block copolymers containing p-PBI and sulphonated PBI (s-PBI) in different ratios for the preparation of membranes, which displayed fuel cell performances at 160 °C with voltages around 0.74 V at 0.2 A/cm^2^. The highest conductivity was found for the 25:75 s-PBI/p-PBI membrane (0.376 S/cm). The same group has also described the preparation of polyphenylquinoxaline-based PBI copolymers via the polyphosphoric acid (PPA) process with proton conductivities up to 0.26 S/cm [112]. Using the PPA process, a series of pyridine-containing *m*-PBI copolymers were used for the preparation of membranes with enhanced mechanical properties, which displayed a proton conductivity of 0.16 S/cm at 160 °C [113]. Fuel cell performances of these membranes were similar to those of *para*-PBI and long-term stability showed these copolymers maintained a consistent fuel cell voltage of 0.6 V at 0.2 A/cm^2^ for over 2300 h. In a later study, the long-term stability was extended to more than 8000 h [114]. A series of PBI-based block copolymers consisting of phosphophilic PBI and phosphophobic non-PBI segments were synthesized via coupling of ortho-diamino terminated *meta*-PBI telechelic macromonomers and carboxylic acid end-capped poly (arylene ether) telechelic macromonomers (Figure 12). The block copolymer PBI-fluorinated poly (arylene ether) displayed a conductivity up to 0.11 S/cm after doping with concentrated PA, but having low mechanical properties [115]. The fuel cell performance showed a 20 μV/h degradation rate when operating at 0.2 A/cm^2^ over 2000 h with an initial 0.58 V at 0.2 A/cm^2^.

Maity and Jana reported the synthesis of a series of *meta*-PBI-block-*para*-PBI segmented block copolymers with different block lengths by the condensation reaction of diamine-terminated *meta*-PBI and acid-terminated *para*-PBI oligomers [116]. The maximum proton conductivity for this block copolymer was 0.11 S/cm at 160 °C, displaying a block structural influence on the proton conductivity. Pan and coworkers synthesized a series of sulfonated polybenzimidazole multiblock copolymers containing pyridine rings via the condensation polymerization of a dicarboxyl monomer containing pyridine, 3,3′,4,4′-tetraaminobiphenyl, and 4-aminobenzoic acid in polyphosphoric acid. Next, membranes were prepared via the generally used solution casting method and subsequently doped with phosphoric acid [117]. The prepared membranes displayed an improved thermal and oxidative stability, reaching proton conductivities up to 0.23 S/cm at 180 °C.

**Figure 13:**
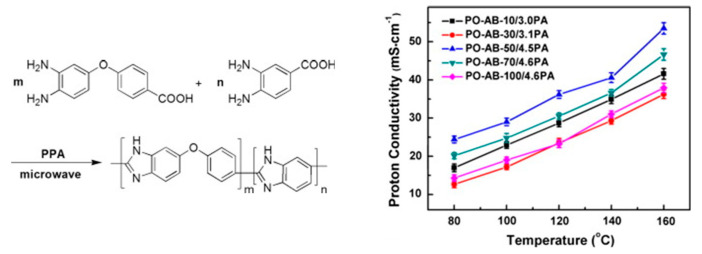
Synthetic route of phenoxyl-containing ABPBI (**left**) and (**right**) proton conductivity of PO-AB-x membranes as a function of temperature (PO-AB-x, x refers to the molar feed percent of 4-(3,4-diaminophenoxy)benzoic acid (DPBA) in total monomers). Reproduced from [118] with permission of Elsevier.

Co-polymers of ABPBIs containing phenoxy in the main chain were synthesized by Wang and coworkers via the solution casting method. The copolymers were synthesized by copolymerization of 3,4-diaminobenzoic acid (DABA) and 4-(3,4-diaminophenoxy)benzoic acid (DPBA) under microwave irradiation. The polymeric membranes displayed an improved stability in organic solvents with proton conductivities up to 0.05 S/cm at 160 °C for the PA-doped membranes (Figure 13); however, a low tensile strength of ∼2 MPa was reached after acid doping [118]. Kim et al. prepared cross-linked copolymer membranes of polybenzimidazole and polybenzoxazine [119]. The copolymerized membranes showed a maximum proton conductivity of 0.12 S/cm at 150 °C under anhydrous conditions. Fuel cell tests showed operating voltages of 0.71 V at 0.2 A/cm^2^, with an excellent long-term durability up to 2000 operating cycles.

## 5. Composite Membranes

Development of the composite PBI membranes, i.e., appropriate combination of inorganic-organic hybrid materials and polymer matrix, allowed significant improvement in the proton conductivity [120]. These composite materials are very attractive since they take advantage of the properties of inorganic and polymeric materials. Moreover, introduction of various inorganic or organometallic fillers into the same polymer matrix results in formation of novel materials. Different fillers were proposed for improving the mechanical integrity of PBI composite membranes. As a first generation, such fillers as silica (SiO_2_) [121], titanium (TiO_2_) [122], zirconia (ZrO_2_) [123], and carbon materials [124] were reported to improve the dimensional stability, mechanical properties and gas permeability of PBI composite membranes. Recently, a second generation of acid functionalized fillers have received growing attention due to the mutual contribution to the proton transport. In this regards, various carbon-based substrates including graphene oxide (GO) [125,126] and carbon nanotubes (CNTs) [127], ionic liquids (ILs) [128], metal organic frameworks (MOFs) [77], nanofibers [129], among others were reported.

The properties of PBI composite membranes classified by the type of filler are outlined as follows.

### 5.1. PBI/Inorganic Composite Membranes

In order to address limitation issues in PA doped PBI membranes, such as low mechanical properties caused by high doping level and acid leaching from the membrane in extreme temperatures, the most widely used approach is the incorporation of an inorganic filler. Introducing various inorganic fillers greatly contributes to the improvement of the membrane behavior and its intrinsic ability to conduct protons. It was found that the addition of inorganic material to PBI to obtain composite or nanocomposite membranes allowed to improve the proton conductivity, water/PA uptake and retention, durability, high mechanical, thermal, and chemical stability at high temperatures, as well as overall fuel cell performance [130,131].

#### 5.1.1. Metallic Oxides

**Figure 14:**
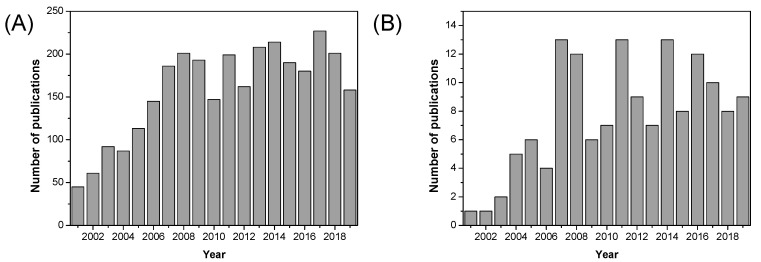
Number of publications in the period of 2001–2019 indexed in the Web of Science: (**A**) keywords: metallic oxide AND fuel cell; and (**B**) keywords: metallic oxide AND fuel cell AND proton exchange membrane. Source: www.webofknowledge.com.

Metallic oxide fillers such as SiO_2_, TiO_2_, and ZrO_2_ were introduced into PBI matrices and some improvements in terms of dimensional stability, mechanical properties, and gas permeability of such formed composite membranes were observed. Over the past decade, a significant increase in the use of fillers based on metallic oxide compounds has been observed for fuel cell applications, as can be seen in Figure 14. As shown in Table 7, proton conductivity values close to 0.12 S/cm can be reached using metallic oxides as inorganic fillers in composite PBI membranes.

Pu and coworkers [132] prepared PBI/SiO_2_ composites with up to 15% SiO_2_ content; the membranes were thermally stable up to 600 °C and generally exhibited better mechanical properties than the PBI membranes of the same structure, but without the filler. A decrease in the tensile strength was observed only at higher SiO_2_ content. The membranes had proton conductivity of 3.9 × 10^−3^ S/cm at 180 °C. Later, Devrim and collaborators [133] prepared PBI/SiO_2_ membranes that were tested in single cell HT-PEMFC; they exhibited higher proton conductivity of 0.103 S/cm at 180 °C. For comparison, the conductivity of the same PBI membrane without filler was 0.094 S/cm. Many efforts have been devoted to improving the compatibility and membrane properties, studying combinations of various metals or modified metal oxide structures as fillers. For example, high conductivity of 0.31 S/cm was obtained for composite membranes filled with mixed Al-Si. The filler was readily produced by the sol–gel method [134]. The proton conductivity and MEA performance of PBI/Al-Si composite were improved with the increasing Al-Si concentration, but the mechanical properties were not desirable, as they got worse. Lysova and coworkers prepared a polybenzimidazole based on 3,3′,4,4′-tetraaminodiphenyloxide and 3,3′-bis(p-carboxiphenyl)phtalide (PBI-O-PhT) and formed composite membranes using SiO_2_ or ZrO_2_ added by two different methods: (1) addition of preliminarily synthesized particles in situ during the polymer synthesis and (2) addition during the casting [135]. It was shown that the modification by zirconia with the in situ method improved the ionic conductivity better than the modification by silica. In the case of the casting method, better results were obtained for silica modification. Zhang and coworkers prepared a membrane using an OPBI polymer and zirconium phosphate (Zr(HPO_4_)_2_·*n*H_2_O, ZrP) [136]. The PBI/ZrP exhibited excellent mechanical strength with 10 wt.% ZrP and showed the highest proton conductivity of 0.192 S/cm at 160 °C under anhydrous condition. Lobato and coworkers prepared membranes PBI by casting in the presence of 2 wt.% TiO_2_, and the fuel cell with this composite showed better performance compared with the fuel cell with the standard PBI membranes, achieving 1000 mW/cm^2^ at 175 °C [137]. The study of PBI composites using mixed Fe_2_TiO_5_ oxides was also performed [138]. According to the Lewis acid–base theory, the main cations Ti^4+^ and Fe^3+^ are classified as hard acids [139]. This means that they may easily react with OH of water. Additionally, it was suggested that, when Fe^3+^ cations were placed near Ti^4+^ cations, as occurred in the Fe_2_TiO_5_ structure, their acidity was increased. Therefore, Fe_2_TiO_5_ single-phase nanoparticles were more hydrophilic than both TiO_2_ and Fe_2_O_3_ nanoparticles separately [140]. The PBI/Fe_2_TiO_5_ membranes showed a higher acid uptake and proton conductivity compared with the pure PBI membranes. The proton conductivity of 0.078 S/cm at 180 °C was observed for the PBI/Fe_2_TiO_5_ containing 4 wt.% of nanoparticles.

The composite membranes prepared from silicotungstic acid supported on silica (SiWA-SiO_2_/PBI) were studied by Staiti [141]. The silica was necessary to entrap the acid, averting its dissolution in water, and to improve the proton conduction. The best result was obtained for the membrane with 50 wt.% of inorganic material; it was mechanically stable and gave a proton conductivity of 1.2 × 10^−3^ S/cm at 160 °C and 100% RH, while the membranes prepared with pure silicotungstic acid had a conductivity of 2.23 × 10^−3^ S/cm under the same conditions.

PA-doped PBIs containing inorganic proton conductors such as zirconium phosphate (ZrP) (Zr(HPO_4_)_2_·*n*H_2_O), phosphotungstic acid (PWA) (H_3_PW_12_O_40_·*n*H_2_O), and silicotungstic acid (SiWA) (H_4_SiW_12_O_40_·*n*H_2_O), or boron (BPO_4_), were investigated by Di et al. [142]. It was concluded that the conductivity of these composite membranes depended on the acid doping level, RH, and temperature, similar to simple PA-doped PBIs. The conductivity was found to be insignificantly higher for the inorganic composite membranes.

Xu et al. [143] presented different PBI-based inorganic–organic composite membranes from Cs substituted heteropolyacids (CsHPAs) with the intention to build MEA for application at intermediate and high temperatures. The CsHPA/PBI membranes loaded with H_3_PO_4_ had much higher conductivity than that of PA-doped PBI. It was observed that conductivity increased with an increase of the filler in the composite. The membrane of 30% CsPOMo/PBI with a doping level of 4.5 exhibited conductivity as high as 0.12 S/cm at 150 °C and anhydrous conditions, and additionally demonstrated excellent mechanical behavior with a strength of 40 MPa. The performance of CsPOMo/PBI/H_3_PO_4_ membranes in a H_2_/O_2_ single fuel cell was also very good, holding a power density of around 0.6 W cm^−2^ with oxygen at atmospheric pressure.

**Figure 15:**
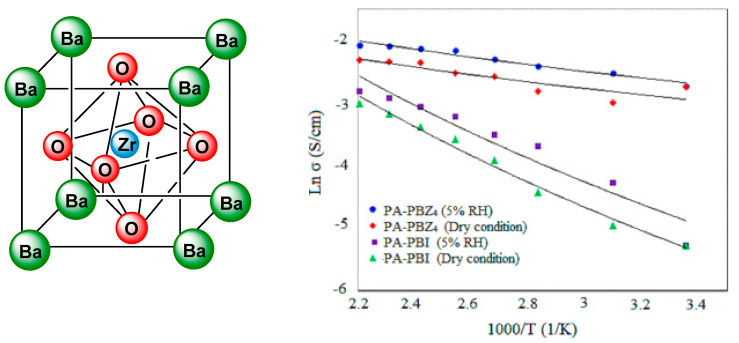
Structure of BaZrO_3_ nanoparticles and Arrhenius plot of proton conductivity for PA-PBI and PA-PBZ_4_ membranes, where Z_4_ means the content of perovskite is 4 wt.%. Reproduced from [144] with permission of Elsevier.

Hooshyari et al. [144] studied the behavior of PBI-BaZrO_3_ (PBZ) nanocomposite membranes for HT-PEMFC; their results showed that the water uptake, acid doping level, and proton conductivity of the PBZ were higher than that of the virgin PBI membrane owing to the presence of BaZrO_3_ perovskite nanoparticles (Figure 15). The proton conductivity observed for these composites containing 4 wt.% of the nanofillers was around 0.125 S/cm at 180 °C, and the performance in a mono fuel cell produced a power density at 0.5 V and 180 °C of 0.56 W/cm^2^, below a 5% RH with a current density of 1.12 A/cm^2^. These results indicate that such membranes are excellent candidates for HT-PEMFC.

Akbar et al. studied the composite membranes using perovskite-type SrCeO_3_ nanoparticles for improving their properties at high temperatures for the application in HT-PEMFC [145]. The PA-doped PBI/SrCeO_3_ membranes showed higher acid uptake (190%), excellent proton conductivity (0.105 S/cm at 180 °C), and better thermal stability at 8 wt.% of SrCeO_3_ (PSC_8_) content in comparison with the pure PBI membrane. The performance of the PSC_8_ nanocomposite membrane showed a 0.44 W/cm^2^ power density and 0.88 A/cm^2^ current density at 0.5 V and 180 °C. The results obtained in these studies clearly demonstrated the enhanced potential of the PSC_8_ as PEM for high temperature proton exchange membrane fuel cells.

#### 5.1.2. Metalcarboranes

**Figure 16:**
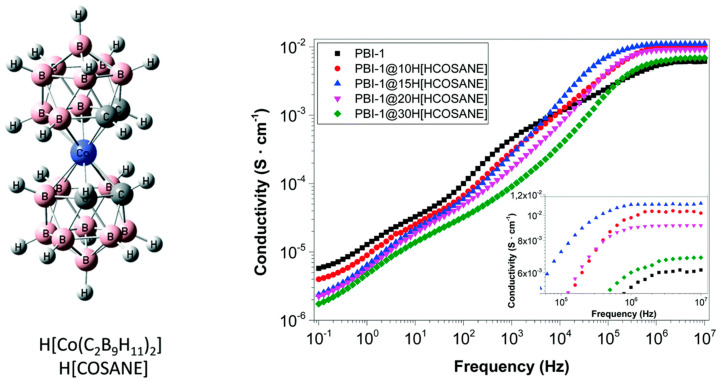
Ball and stick view of H[COSANE] (**left**) and Bode diagram for composite membranes of PBI containing 15 wt.% of H[COSANE] (**right**). Reproduced from [148] with permission of the Royal Society of Chemistry.

Our group, in collaboration with the group of F. Teixidor, has also studied a family of inorganic fillers based on sandwich compounds of molecular formula M[Co(C_2_B_9_H_11_)_2_] (M = Li+, Na+, H+), also named M[COSANE] (Figure 16). These compounds are highly stable anionic materials with a very low charge density. In 2017, we investigated the temperature dependence of the proton conductivity of the cobalt salt H [COSANE] under wet and dry conditions. We observed that conductivity was strongly dependent on the relative humidity and was higher in H[COSANE] than in other metallacarboranes from the same family such as Na[COSANE] or Li[COSANE]. In this regard, the observed conductivity of H[COSANE] was similar to that of other PBI membranes containing carboxylic groups and inorganic fillers, reaching values up to 0.01 S/cm [146]. Recently, we have used these fillers in the preparation of composite proton exchange membranes based on PBI. In a cation study, using H[COSANE], Li[COSANE], and Na[COSANE] fillers, the conductivity of the composite membranes followed the trend σ(PBI@H[COSANE]) > σ(PBI@Na[COSANE]) > σ(PBI@Li[COSANE]), reaching values close to 0.001 S/cm [147]. The electrochemical impedance spectroscopy results showed that conductivity increased with temperature and is higher for H^+^ than for Li^+^ and Na^+^ for all temperatures under study. The temperature dependence of the conductivity of the composite was followed by a typical Arrhenius behavior with two different regions: (i) between 20 and 100 °C and (ii) between 100 and 150 °C, whose activation energy values are given in Table 8. The pristine PBI membranes show that the conductivity strongly begins to fall down, which may be because of the hydration of the membrane when the temperature is higher than 100 °C. However, for the PBI composite membrane, the authors observed a second behavior between 100 and 150 °C, where the conductivity tends to increase with a different slope compared with the first interval up to 150 °C. At temperatures above 150 °C, in composite membranes, conductivities decreased when temperature increased. This is possible owing to the solvent evaporation temperature used in membranes’ preparation. The evaporation temperature of solvent (DMAc) is about 160 °C, which could explain why membrane conductivity was found to be reduced above this temperature value.

In a recent study, the effect of the H[COSANE] concentration in three different polymeric matrices based on the PBI structure, PBI, OPBI, and *o*-6F-PBI have been investigated [148]. All prepared membranes displayed excellent proton conductivities higher than 0.03 S/cm above 140 °C, reaching a maximum when the amount of H[COSANE] was 15 wt.%.

### 5.2. Graphene Oxide, Carbon Nanotubes, and Others

**Figure 17:**
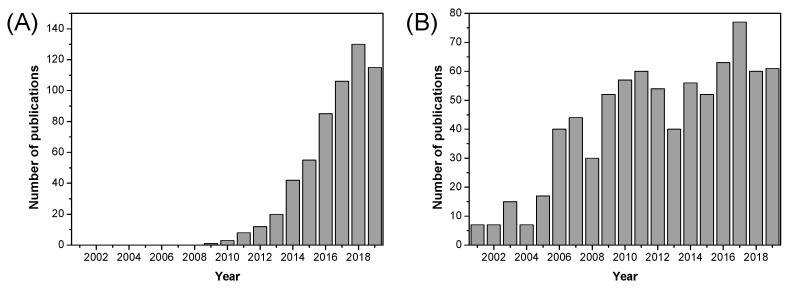
Number of publications in the period of 2001–2019 indexed in the Web of Science: (**A**) keywords: graphene oxide AND fuel cell AND proton exchange membrane; and (**B**) keywords: carbon nanotube AND fuel cell AND proton exchange membrane. Source: www.webofknowledge.com.

Another type of filler that has been attracting attention for the last two decades is the family of graphene nanomaterials, mostly in the form of nanotubes or graphene oxide (GO). In particular, GO has received special interest because of the unique combination of its properties, as can be shown by the number of publications over the last decade (Figure 17). GO is formed from the oxidation of graphite and comprises 2D carbon sheets, but is decorated with oxygen-containing functionalities on the edges (hydroxyl, carbonyl, and carboxyl groups) and on the surface (hydroxyl and epoxide groups) [149,150].

Thus, GO maintains all exceptional properties of graphene, but in contrast to graphene, GO is easily dispersible in water, organic solvents, or polymer matrices owing to the presence of the oxygen functionalities, whereas graphene has a strong tendency to agglomerate. The use of GO as a precursor is the most reliable and effective approach for preparing graphene-based polymer composites. GO typically preserves the layer structure of the graphite, but the layers are buckled and the interlayer spacing is much larger than the graphite [151]. GO is an electronic insulator with differential conductivity; however, when GO is added into a polymer matrix, the protons presented in the membrane interact with GO, and this promotes proton conductivity on the composite membrane [152]. Owing to its unique properties, GO has found tremendous applications in a diverse range of fields, such as gas barrier nanocomposites [153], water treatment [154], stimuli-responsive materials [155], energy storage as supercapacitors [156], lithium ion batteries [157], and stretchable electronics [158].

The presence of GO in the membrane provides the advantages of excellent mechanical properties, large specific surface area, and as high as 0.01 S/cm inherent proton conductivity. Therefore, the introduction of GO into the PBI polymer matrix helps to improve the acid doping and proton conductivity, and prevent acid leaching. Besides, GO allows improving the performance and increasing the durability of HT-PEMFC. As shown in Table 9, proton conductivity values close to 0.17 S/cm can be reached using GO as a filler in PBI membranes.

Üregen and coworkers prepared and studied a PBI/GO nanocomposite with different weight percentage GO loading [38]. The maximum proton conductivity of 0.17 S/cm at 165 °C was observed for the membrane with 2 wt.% GO content. The membrane performance in the HT-PEMFC system showed that it was significantly improved in comparison with the pristine PBI membrane under dry conditions. In the last years, attempts to apply GO modified with sulfonated, phosphonated, and other groups have been made in order to improve proton conductivity, as well as dispersion and compatibility between the filler and polymeric matrix. Yang and coworkers prepared a PBI composite membrane with GO bearing triazole groups (TrGO) [159]. The presence of triazole functionality allowed improving the compatibility with the polymer. The PBI/TrGO membranes with 1.2 wt.% of TrGO were much more uniform and homogeneous than the PBI/GO membranes. The last ones were quite heterogeneous and demonstrated obvious phase-separation. The tensile strength of the PA doped composite membranes was 12.6 MPa, which was much higher than that of the PBI membrane with a similar acid doping level (ADL) of around 12. Moreover, the high proton conductivity of 0.135 S/cm at 180 °C was achieved in this membrane.

**Figure 18:**
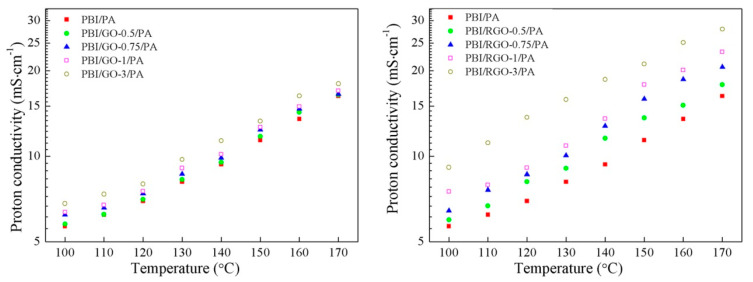
Temperature dependence of proton conductivity of PBI/GO/PA (**left**) and PBI/RGO/PA (**right**) membranes. Reproduced from [126] with permission of John Wiley and Sons.

Cai and coworkers prepared sulfonated GO (SGO), using the ^60^Co γ-ray radiation grafting method, which was then added into PBI via solution-casting [126]. The sulfonic acid groups in SGO were able to form stronger interactions with the –N = or −NH groups of the benzimidazole ring than the oxygen-containing groups of the non-modified GO, and thus SGO was well dispersed in the polymer even with the SGO content of 1 wt.% (PBI/SGO-1%). Additionally, the composite exhibited much better mechanical properties, with a tensile strength of 133.1 MPa, an elongation at break of 36.4%, and a tensile modulus of 2134 MPa. These values increased in 32.0%, 220%, and 33% compared with those for pure PBI membrane, respectively. However, when SGO content was >1%, low dispersion in the polymer matrix led to a reduction in the mechanical properties of the membranes. The proton conductivity of PBI/SGO-1%/PA was 0.023 S/cm at 170 °C under dry conditions (Figure 18). Then, composites of 2,6-pyridine functionalized PBI with highly dispersible phosphoric acid functionalized GO (PGO) were proposed as candidates for durable performance at elevated temperature (PBI-Py/PGO) applied in fuel cells [116]. The incorporation of 1.5 wt.% of PGO in the membrane attained the highest conductivity value of 76.4 × 10^−3^ S/cm at 140 °C (compared with 19.6 × 10^−3^ S/cm for PA doped PBI-Py membrane under similar conditions). In addition, the durability of the proton transport was significantly improved in the PGO containing membranes.

Carbon nanotubes (CNTs) have been shown to be promising materials for various applications, such as biological and biomedical research [160,161], environmental science [162], catalysis [163], fabrication of composite materials [164], microelectronics [165], solar cells [166,167], electronic components [168,169], energy storage [170], hydrogen storage [171], and so on. The PBI-based composed membranes with CNTs (PBI/CNT) were investigated and their properties were compared with the plain PBI membrane. The proton conductivity of PA-doped PBI and PBI/CNT was 0.063 and 0.074 S/cm, respectively, at 180 °C [172].

**Figure 19:**
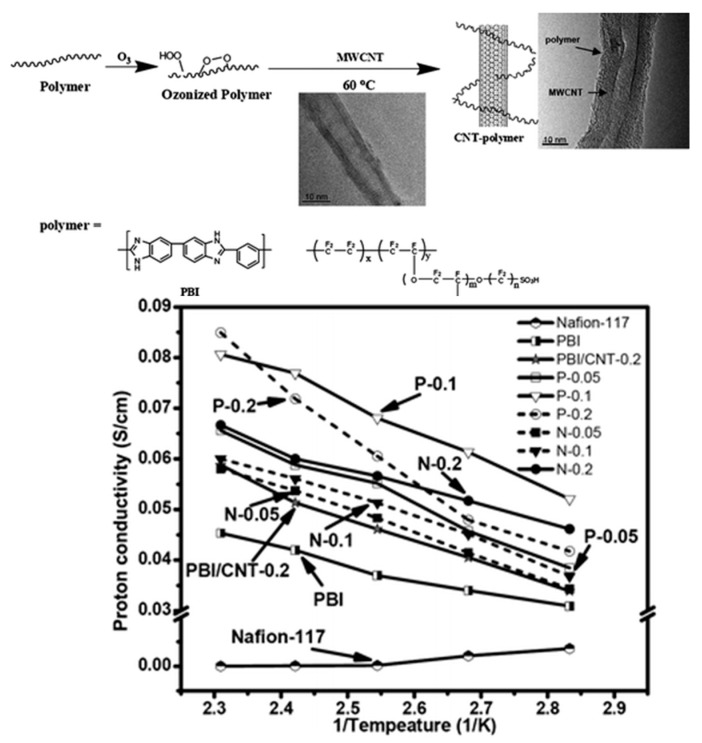
Schematic representation of preparation of sulfonated Nafion^®^- and PBI-functionalized multiwalled carbon nanotubes (MWCNTs) (**top**) and proton conductivity of different composite membranes at 80–160 °C without humidification (**bottom**). Reproduced from [174] with permission of the Royal Society of Chemistry.

Liu and co-workers prepared PBI-functionalized multiwalled carbon nanotubes (MWCNTs) through an ozone mediated process [173] and used as fillers in the preparation of PBI/MWCNT nanocomposite membranes [174]. In the preparation of PBI/MWCNT nanocomposite membranes using the conventional solution casting method, MWCNT/PBI were well dispersed in the PBI solution. Interestingly, the addition of the functionalized MWCNTs enhanced the thermal and mechanical properties of the composite membranes, increasing the Young’s moduli and tensile strength 70% and 75%, respectively, with respect to pristine PBI membrane. Regarding the PA-doped PBI nanocomposite membrane containing 0.2 wt.% of MWCNT–PBI, it had a proton conductivity of 0.08 S/cm at 160 °C under anhydrous conditions, compared with the proton conductivity of 0.045 S/cm of the PA-doped PBI membrane (Figure 19). The authors attributed the high proton conductivity to the relatively high acid uptake level of PBI composite membranes and proton migration under anhydrous conditions was described to be mainly based on a Grotthuss-type mechanism. In studies of performance in single cell tests at 150 °C, the MWCNT/PBI composite membranes demonstrated maximum power densities of 600 mW/cm^2^. These values are much higher than that found with a pristine PBI membrane (530 mW/cm^2^) and lower than that of MWCNT/Nafion^®^ nanocomposite membranes containing the same amount of MWCNT, whose value was 700 mW/cm^2^.

On the other hand, the PBI nanocomposite membranes filled with phosphonate functionalized carbon nanotube (P-MWCNT) were also prepared and their properties were studied. It was shown that the two properties of key importance for PEM—the proton conductivity and mechanical stability above 100 °C—were improved [175]. It turned out that P-MWCNTs, being incorporated into the PA-doped PBI matrix, organized in domain-like structures. The enhanced performance was attributed to the formation of proton conducting networks that formed along the sidewalls of P-MWCNTs with a domain size of 17 nm.

In 2013, Hsu and co-workers prepared PBI composite membranes containing carbon nanotubes with different functional groups, which were studied for proton exchange membrane fuel cells [176]. Two approaches were employed in the functionalization of MWNTs. The first functionalization involved non-covalent modification by an in situ radical polymerization of sodium 4-vinylbenzenesulfonate with MWNTs to yield MWNTpoly (NaSS) [177]. The second approach involved the covalent modification of COOH-modified MWNTs by reaction with 1-(3-aminopropyl) imidazole through DCC-mediated amide bond formation to afford imidazole-functionalized MWNTs [178]. Next, composite membranes were prepared via the casting method using mixtures of functionalized MWNT and a fluorine containing PBI solution. The PBI composite membranes containing imidazole-functionalized MWNT provided more significant mechanical reinforcement compared with unmodified MWNTs and MWNT-poly (NaSS) membranes, which was attributed to its better compatibility with PBI. For PA doped MWNT-poly(NaSS)/PBI and MWNT-imidazole/PBI composite membranes, the proton conductivities were up to 0.051 and 0.043 S/cm at 160 °C under anhydrous condition, respectively, which were slightly higher than pristine PBI (0.028 S/cm). This enhancement was attributed to the combination of the increase of free volume of the membranes at higher temperatures and the positive correction between volume swelling and acid-doping level.

In 2015, Guerrero Moreno and co-workers prepared composite polymeric PBI membranes filled with 1 wt.% of MWCNTs (with 20 and 140 nm inner and outside diameter, respectively, and 8 μm length) by spin coating [179]. The mechanical stability of the PBI membrane improved upon the addition of the carbon-based materials. The tensile strength of the composite PBI/CNT membrane with 1 wt.% CNTs loading was found to be 32% higher than the pristine PBI membrane. When studying the proton conductivity at 180 °C under anhydrous conditions, the PA doped PBI/MWCNT composite membrane exhibited a conductivity of 0.074 S/cm, slightly higher than that for the PA doped PBI membrane (0.063 S/cm). The authors attributed this enhancement to the higher molar ratio of PA to the polymer-repeat-unit owing to the presence of the CNTs.

### 5.3. Metal Organic Frameworks

Metal–organic structures (MOFs) are another type of commonly used filler used to improve proton conductivity in polymer electrolyte membranes. In recent years, the use of metal–organic structures as fillers in PEMs has attracted interest owing to their high conductivity, which is mainly attributed to their high porosity and the retention of water molecules in their pores [180,181,182,183]. The MOFs are a family of metal-based crystalline porous materials that contain bonding organic units, which can form strong bonds, and lead to the creation of open crystalline structures with regular porous arrangement [184,185,186]. These interesting compounds have been studied for possible different applications such as fuel cells [187], pervaporation [188], nanofiltration [189], gas separation [190], desammonia adsorption [191], and organocatalysis [192]. The introduction of MOFs into organic polymer results in the enhanced thermal chemical stability of the formed composites under harsh conditions, making them suitable for industrial application [193]. For elaboration of these membranes, polymers such as perfulorosulfonic acid [194], sulfonated poly (ether ether ketone) [195], and poly(vinyl alcohol) [196] have been used.

**Figure 20:**
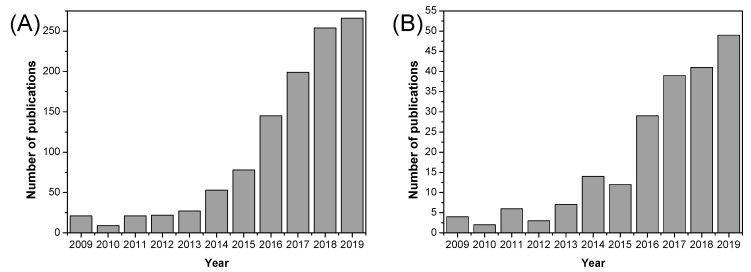
Number of publications in the period of 2009–2019 indexed in the Web of Science: (**A**) keywords: metal organic framework AND fuel cell; and (**B**) keywords: metal organic framework AND fuel cell AND proton exchange membrane. Source: www.webofknowledge.com.

As illustrated in Figure 20, the number of published research articles on MOF containing materials for fuel cell applications has increased significantly over the past decade, especially in recent years (2016–2019). Over the past decade, a diverse range of MOFs have been used as fillers in the preparation of mixed matrix membranes based on Nafion^®^ [197,198,199,200,201], SPEEK [202,203,204,205,206], and other polymeric materials [207,208,209,210,211]; however, their use in PBI polymers is still very scarce.

**Figure 21:**
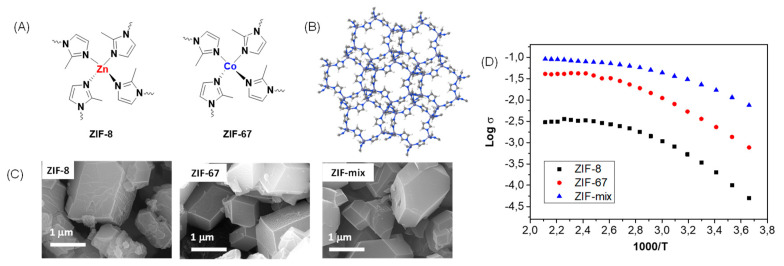
(**A**) Schematic representation of chemical structure of ZIF-8 and ZIF-69. (**B**) Structural representation of ZIF-8. (**C**) Field-emission scanning electron microscopy (FE-SEM) images of ZIF-8, ZI-67, and ZIF-mix. (**D**) Arrhenius plot of phosphoric acid doped PBI composite membranes containing 5 wt.% of ZIFs [77].

In this regard, our group prepared PBI membranes containing the imidazolate zeolites (ZIFs) [77], a subclass of MOFs. ZIFs are materials with zeolitic topology when a M^2+^ tetrahedral divalent metal cation (M = Co, Zn) coordinates to four imidazolate rings, forming a neutral (M(Im)_2_) porous structure of high chemical and thermal stability [212,213]. The conductivity in the ZIF/PBI composite membranes containing 5 wt.% ZIF fillers varied from 0.003 to 0.091 S/cm at 200 °C, depending on the type of ZIF (Figure 21).

### 5.4. Ionic Liquids and Other Conductive Compounds

The use of ionic liquids (ILs) as conductive fillers to replace PA was proposed in an attempt to overcome the disadvantages of PA, such as leaching and conduction instability with the time mentioned above. ILs are purely ionic materials with generally low, below 100 °C, melting temperatures [214]. ILs have found applications in a wide variety of fields including their use as green solvents in organic synthesis [215], catalysis [216,217], extraction, separation [218], supramolecular chemistry [219,220], transport agents [221], pharmaceutical chemistry [222], materials science, and drug sensing [223], among others. ILs have several favorable properties including their temperature stability, non-volatility and non-flammability, rather high ionic conductivity, and reduced environmental impact. So, ILs are considered as promising compounds for the preparation of electrochemical devices [224]. As shown in Table 10, proton conductivity values close to 0.1 S/cm can be reached using ionic liquids as fillers in composite PBI membranes.

**Figure 22:**
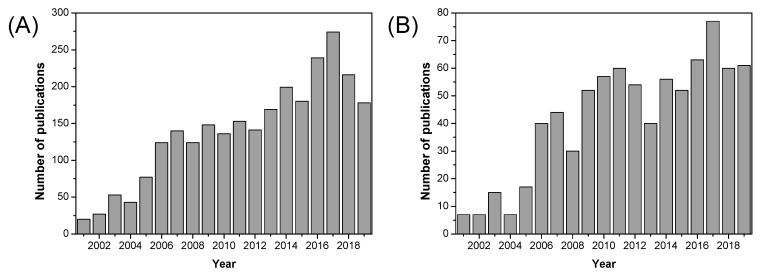
Number of publications in the period of 2001–2019 indexed in the Web of Science: (**A**) keywords: ionic liquid AND fuel cell; and (**B**) keywords: ionic liquid AND fuel cell AND proton exchange membrane. Source: www.webofknowledge.com.

As shown in Figure 22, ionic liquids began to be used in in fuel cells since their discovery at the beginning of the 21st century, and the number of publications of the use of ionic liquids in fuel cells has flourished in the past 15 years.

Wang and coworkers prepared a polymer composite membrane based on fluorine containing PBI and 1-hexyl-3-methylimidazolium trifluoromethanesulfonate (HMI-Tf) as IL [225]. The conductivity of the PBI/HMI-Tf membrane was 0.016 S/cm at 250 °C under anhydrous conditions. Then, Ven and coworkers introduced 1-H-3-methylimidazolium bis(trifluoromethanesulfonyl)imide ([h-mim] Ntf_2_) IL in the PBI support [226]. The resulting membrane showed a proton conductivity of 0.002 S/cm at 190 °C and the thermal stability in the range of 150–190 °C. Recently, our group prepared a series of PEMs based on PBI filled with 1-butyl-3-methylimidazolium (BMIM) bearing different anions (Cl, I, BF_4_, PF_6_, NCS, Br, NTf_2_, BF_4_) [227,228]. Under PA doping conditions, these composite membranes with 5 wt.% of ILs exhibited the highest proton conductivity of 0.098 S/cm at 120 °C when BF_4_ anion was present.

**Figure 23:**
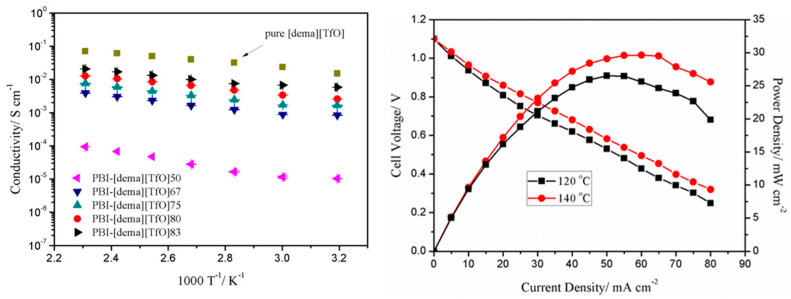
Variation of the conductivity of the ionic liquid (IL) and membranes at different temperatures (**left**) and polarization curve of an anhydrous H_2_/Cl_2_ fuel cell using the PBI/[dema][TfO]_83_ composite membrane at different temperatures (**right**). Reproduced from [229] with permission of the American Chemical Society.

Liu et al. [229] found that the membranes composed of PBI/[dema][TfO]_33_ and PBI/[dema][TfO]_50_ exhibited low conductivity (<10^−4^ S/cm at 160 °C) at a low content of IL in the polymer matrix and reasonably high conductivity (>10^−3^ S/cm at 40 °C) when the concentration of [dema][TfO] in the polymer increased to 83% (PBI/[dema][TfO]_83_). The growth in conductivity at a higher IL content may be explained by enhanced free ionic mobility in the membrane matrix and the formation of well-developed ionic channels. The MEAs built with such composite membranes showed conductivity levels comparable to the data reported for other composite membranes, suggesting that the IL composite membranes have a potential for HT-PEMFC application under anhydrous conditions (Figure 23).

Xipeng Soing et al. [230] prepared a series of poly(oxyphenylene benzimidazole) (OPBI)/ionic liquid (IL) composite membranes mixing the polymer with 1-butyl-3-methylimidazolium tetrafluoroborate ([BMIm]BF_4_). The obtained membranes had high proton selectivity that was useful for application as vanadium redox flow batteries. It was also found that, when the IL content increased, the vanadium resistance and proton conductivity of the membranes increased, obtaining an optimized proton selectivity for OPBI/BF_4_-20 composite membrane. The optimum value was 1.41 × 10^6^ S·min cm^−3^, which was much higher than that of the unmodified OPBI membrane (6.06 × 10^5^ S·min cm^−3^) or a commercialized Nafion^®^ 115 membrane (1.61 × 10^4^ S·min cm^−3^). On the other hand, OPBI/BF_4_-20 exhibited a higher coulombic efficiency (CE, 99.24%), voltage efficiency (VE, 93.10%), and energy efficiency (EE, 92.39%) at 40 mA cm^−2^ than the unmodified OPBI (CE 98.06%, VE 90.67%, and EE 88.86%) and even higher than Nafion^®^ 115 membranes (CE 95.44%, VE 91.75%, and EE 87.57%).

Finally, studies carried out on the PBI-based membranes prepared by covalent crosslinking with triglycidylisocyanurate (TGIC) and doped with highly sulfonated polyaniline (SPAN) showed the good thermal, dimensional, mechanical, and oxidative stability of these membranes applied in MEAs of direct methanol fuel cells (DMFCs) [231]. The relatively low degree of cross-linking allowed high doping level of SPAN and, consequently, the high proton conductivity. The proton conductivities of PBI-TGIC (5%)/SPAN(50%) and PBI-TGIC(10%)/SPAN (50%) were 0.13 and 0.12 S/cm, respectively, at 180 °C and 100% RH; 0.064 and 0.058 S/cm, respectively, at 180 °C and 50% RH; and 0.018 and 0.016 S/cm, respectively, at 180 °C and 0% RH.

### 5.5. Electrospinned Fillers

**Figure 24:**
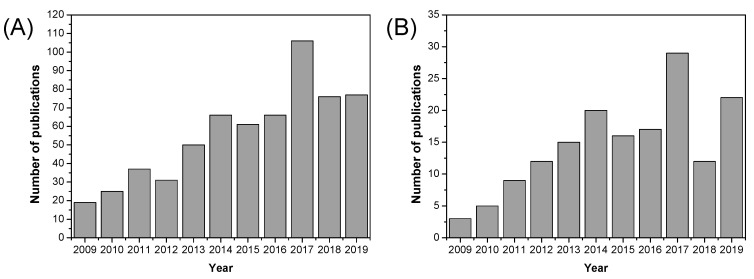
Number of publications in the period of 2009–2019 indexed in the Web of Science: (**A**) keywords: electrospinning AND fuel cell; and (**B**) keywords: electrospinning AND fuel cell AND proton exchange membrane. Source: www.webofknowledge.com.

Among the different approaches to develop novel materials in the fabrication of PEMFC for sustainable energy devices, nanofibrous structured materials have become an efficient alternative in the midst of several fuel concerns. As displayed in Figure 24, the number of publications concerning nanofibrous materials for fuel cell applications is still growing.

Electrospinning has generated considerable interest as a promising method for fabricating nanofiber-based PEMs owing to the specific properties associated with its advanced features, including the high surface area, low density, high porosity, fully interconnected pores, high orientation or alignment of nanofibers, and easy scalability.

PEMs composed of aligned electrospun nanofibers can offer a uniaxial arrangement of the polymer chains in nanofibers, thereby providing better mechanical properties and promoting the formation of interconnected channels, resulting in enhanced proton conductivity [232]. In the field of PEMs, polymers such as sulfonated poly (ether ether ketone) [233,234], polyimide [235], and Nafion^®^ [236,237], among others, have been electrospun into fibers.

Li and coworkers prepared polybenzoxazine (PBz)-modified polybenzimidazole (PBI) nanofibers by the electrospinning process [238]. The nanofibers were crosslinked through the ring-opening addition reaction of the benzoxazine groups. Modification of the PBI composite membranes with the crosslinking PBI nanofibers significantly improves their mechanical properties, acid uptakes, and dimensional stability upon acid doping. The composite membranes showed proton conductivity of 0.17 S/cm at 160 °C under anhydrous conditions, which was about twofold higher than the proton conductivity of the neat PBI membrane. Muthuraja and coworkers prepared different types of membranes from the whole poly (aryl sulfone ether benzimidazole) (SO_2_-OPBI) and from its nanofibers obtained by the electrospinning process [239]. Compared with the traditional PBI, presence of the sulfone and ether linked in the polymeric backbones improved the membrane flexibility and its resistance towards radical oxidation, as noted above. Nanofiber SO_2_-OPBI membrane reached proton conductivity of 0.067 S/cm, which was higher than that of dense SO_2_-OPBI (0.033 S/cm) and PBI membranes (0.008 S/cm) at 160 °C. As a result, the acid doped SO_2_-OPBI membranes showed better chemical strength and higher proton conductivity.

**Figure 25:**
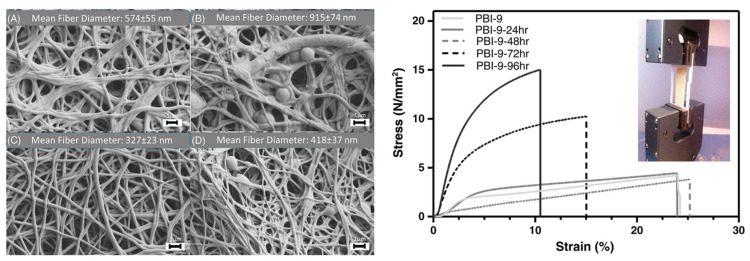
(**Left**) Scanning electron microscope micrographs of (**A**) PBI-9–24 h, (**B**) PBI-9–48 h, (**C**) PBI-9–72 h, and (**D**) PBI-9–96 h phosphoric acid–doped polybenzimidazole (PBI) nanofibers and (**right**) stress–strain curves of undoped and PA doped during 24, 48, 72, and 96 h electrospun membranes. Reproduced from [240] with permission of John Wiley and Sons.

Jahangiri and coworkers produced PBI electrospun nanofiber of 170 nm diameter [240]. Immersion into PA for 72 h led to a highest proton conductivity of 0.123 S/cm, whereas the conductivity of 96 h doped PBI mats decreased. Tensile strength of the membranes was found to increase with doping level, whereas the strain at break (%) decreased because of the brittle nature of the formed network (Figure 25).

**Figure 26:**
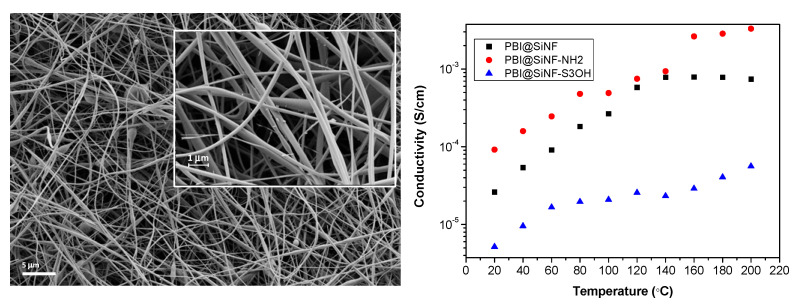
FE-SEM images of SiO_2_ nanofibers and temperature dependence of composite PBI membranes with neutral (PBI@SiNF), basic (PBI@SiNF–NH_2_), and acidic groups (PBI@SiNF–SO_3_H) [76].

Our group has also contributed to the field of nanofiber materials through the preparation of PBI composite membranes containing SiO_2_ nanofiber mats [76]. The nanofiber materials were fabricated via the electrospinning process and later functionalized with terminal neutral, acidic, and basic groups using silane chemistry. This surface functionalization was characterized by X-ray photoelectron spectroscopy (XPS), which is a widely used surface characterization technique [241,242,243]. The different functionalized nanofiber mats were embedded into a PBI matrix to fabricate composite membranes with enhanced chemical and thermal stability. Among the diverse composite membranes, those containing nanofibers with basic groups displayed higher conductivity with values up to 0.003 S/cm at 200 °C without phosphoric acid doping (Figure 26). As shown in Table 11, these membranes displayed lower conductivity than the previously reported membranes as they were used in undoped conditions.

## 6. Conclusions

In this review, an extensive outlook of the recent developments on composite membranes based on PBI for HT–PEMFC applications is given. The most common approach to increase proton conductivity in PBI membranes is based on the phosphoric acid doping, which enhances proton conductivity of these polymeric membranes up to 0.2 S/cm. However, degradation of the membrane at high temperatures and acid leaching are drawbacks that hamper their use as HT-PEMFCs. The use of alternatives based on the addition of fillers into the polymeric matrix can help to overcome these problems. As shown, proton conductivity values close to those of the commercially used Nafion^®^ membranes can be reached using a wide diversity of materials used as fillers in PBI membranes. These fillers include carbon-based materials such as graphene oxide and carbon nanotubes, nanofibers obtained by electrospinning methods, inorganic fillers such as metal oxides and heteropolyacids, and organic fillers such as ionic liquids or metal organic frameworks. Other alternatives are based on the modification of PBI structure by synthetic methods or co-polymerization strategies, which can increase the proton conductivity and retain phosphoric acid more efficiently. Despite that high conductivities have been reported by these methods, phosphoric acid leaching remains a problem to be solved in the next decade.

Fuel cell technology is currently receiving much attention from researchers as well as from the industry because it can reduce the costs of the organic and inorganic fillers such as metal organic frameworks, ionic liquids, nanofibers, and carbon-based materials. The introduction of inorganic fillers into the polymer matrix of PBI in order to form novel composite membranes was reported to improve the dimensional stability, mechanical properties, and gas permeability of PBI composite membranes.

The effective design of composite membranes based-PBI doped with phosphoric acid for high performance PEMs for fuel cells has been demonstrated with higher performance and durability. The composite membranes based on PBI formed with nanofibers and organic and inorganic fillers show good mechanical properties and solvent-resistance, proving the latter to be effective additives for the preparation of reinforced PBI composite membranes through a solution process enhancing the formation of long-range proton-conductive pathways in the PBI membranes. The acid-uptake levels, dimensional stability upon acid-doping, and proton conductivity of the PBI-based membranes through retention of phosphoric acid have been significantly enhanced by the formation of composite membranes with cross-linked PBI. Cross-linking plays an important role in forming additional networks in PBI, despite that it generally weakens the interaction between the filler and polymer chain; however, cross-linking reinforces the polymer chain with additives producing more novel rigid materials. On the other hand, plasticizers can form composites membranes with high flexibility, reducing hydrophilic or hydrophobic properties (depending on the nature of the plasticizers), and even increasing the proton conductivity. The addition of plasticizers has also increased the amorphous phase in PBI composites.

The use of additives involves a wide variety of technologies in polymer technology, with a great field of materials engineering to be applied in energy existing today. The focus of this review has been the use of additives in PBI membranes to facilitate PA doping to be durable over prolonged cyclic usage and, in the long term, especially to obtain high performance and durability in HT-PEMFC with low costs. The reported membranes have been studied from their physical structure and morphology as well as their chemical and electrochemical performance. As per our findings, most of the previous work found in the literature reported that the presence of additive material in a polymer matrix enhances the properties compared with the neat polymer. However, most of the synthesized membranes display poor performance in a single fuel cell in the presence of additives with a diminution in terms of proton conductivity and mechanical strength owing to some problems such as the agglomeration, swelling, and interaction filler polymeric PBI matrix, among others. Consequently, there is a strong need to overcome these drawbacks and reach an equilibrium between proton conductivity and thermal and mechanical stability in the composite membrane.

The challenges and opportunities of composite membranes of PBI are still growing as their impact on the research and development (R&D) industry is under continuous growth, as the demand increases every year following that of devices operating at moderate and high temperatures. Thus, the design of novel materials is of crucial interest when providing major durability and strength of PBI. However, a crucial requirement they need to fulfill to be applied as fuel cells and as super capacitors is the need for high conductivity and long-term durability. For this purpose, as mentioned above, the use of PA doped membranes can be a strong limitation and, at the same time, an advantage, if the natural route is applied when synthesizing the additive. Nonetheless, there are still some aspects that need to be improved on these composite membranes based on PBI, such as reducing the degradation rates of the polymeric membranes present owing to the operation at high temperatures. It is also desirable to design new PEMs with enhanced chemical stability towards peroxide and radical attacks, as well as increase the retention factor of phosphoric acid to reduce the loss of the electrolyte, and maintain the proton conductivity for extended periods of time.

The market for polymer electrolyte membrane fuel cells is expected to grow at a compound annual growth rate (CAGR) of 15.28% during the next five years. Major factors driving the market are increasing R&D activities for energy applications, which has driven to various technological advantages, such as high power density, decrease in the time to refuel, longer storage durability, and increase of life-cycles of PEM fuel cells over alternatives such as Li-ion batteries; in addition, efforts have been focused on the design of PEM fuel cell-powered vehicles with the help of government incentives and policies. However, the current cost of PEMFC technology is still relatively high, a substantial limitation to overcome. In the next years, attempts have to be oriented towards getting around this important barrier, which can help to successfully implement this green technology in commercial usage for stationary and transportation, among others [244].

In the past few years, considerable progress has been made through the commercialization of HT-PEMFC technology, and it has emerged as an attractive alternative to other kinds. As an example, commercially available Advent PBI MEAs, based on PBI, possess excellent thermal and oxidative stability, and can operate at 120 to 180 °C using phosphoric acid as the electrolyte. Among the advantages, it is worth mentioning that they do not need water for conductivity and can reach proton conductivities up to 0.1 S/cm with a proven lifetime of 20,000 h [245]. The elevated operating temperature leads to important advantages, making them potential candidates as a near-future environmentally friendly technology.

## Figures and Tables

**Table 1:** Proton conduction activation energy calculated by polybenzimidazole (PBI)-triglycidyl isocyanurate (TGIC)/sulfonated polyphosphazene (SPOP) composite membranes.

Polymer	E_a(RH 100%)_ kJ/mol	E_a(RH 50%)_ kJ/mol	E_a(RH 0%)_ kJ/mol
PBI-TGIC(5%)/SPOP(50%)	12.7	19.5	24.3
PBI-TGIC(10%)/SPOP(50%)	14.1	20.8	25.2
PBI-TGIC(5%)/SPOP(40%)	16.2	22.1	26.7
PBI-TGIC(10%)/SPOP(40%)	18.6	23.5	27.1

**Table 2:** Proton conduction activation energy calculated by APBI-dicationic ionic liquid (pr(mim)_2_Br_2_) (DIL)/mesoporous silica (MDA) composite membranes. PA, phosphoric acid.

Polymer	PA/PBI_25 °C_	σ (S/cm)	E_a_ (kJ/mol)
APBI	7	0.051	21.20
APBI-DIL_4_	10.99	0.142	13.56
APBI-DIL_4.5_-MDA_0.5_	13.67	0.182	----
APBI-DIL_4.5_-MDA_1.5_	15.32	0.224	8.87
APBI-DIL_4.5_-MDA_2.0_	15.09	0.221	----

**Table 3:** Proton conduction activation energy calculated by PBI doped with different acids. HPW, phosphotungstic acid.

Polymer	σ_DC_ 140 °C (S/cm)	E_a_ (kJ/mol)
PBI	2.5 × 10^−12^	53 ± 2
PBI–PA 1M	2.5 × 10^−3^	25 ± 3
PBI–PA 14 M	5.3 × 10^−2^	11.6 ± 0.7
PBI–phytic acid	2.6 × 10^−4^	25 ± 2
PBI-HPW	1.9 × 10^−11^	31 ± 3

**Table 4:** Activation energies for the PBI composite membranes in wet and dry conditions for the temperature interval 20–90 °C.

Polymer	E_a(wet)_ kJ/mol	E_a(dry)_ kJ/mol
PBI	55.6 ± 0.8	75 ± 3
PBI-SiNF	12.7 ± 0.4	72 ± 3
PBI-SiNF-NH_2_	10.7 ± 0.3	56 ± 2
PBI-SiNF-SO_3_H	25 ± 1.5	123 ± 10

**Table 5:** Activation energies for the PBI composite membranes along the temperature interval 20–90 °C. ZIF, zeolitic imidazolate framework.

Polymer	E_a_ kJ/mol
PBI	36 ± 2
PBI−ZIF-8	33 ± 2
PBI−ZIF-67	30 ± 2
PBI−ZIF−mix	19 ± 1.4

**Table 6:** Activation energies for the PyPBI-phosphonated graphene oxide (PGO) composite membranes doped PA.

Polymer	E_a(dry)_ kJ/mol
PyPBI	22.8
PyPBI-PGO_1.0_	18.2
PyPBI-PGO_1.5_	18.0

**Table 7:** Composite membranes of polybenzimidazole derivates with inorganic materials.

Polymer	Filler	wt.%	σ_DC_ (S/cm)	T (°C)	Doped	Ref
PBI	---	---	10^−12^	160	---	[64]
PBI	SiO_2_	15	0.004	180	---	[132]
PBI	SiO_2_	5	0.103	180	---	[121]
PBI	Al-Si		0.310		---	[134]
PBI-O-PhT	ZrO_2_	-	0.162	180	D	[135]
OPBI	α-ZrP	10	0.192	160	---	[136]
PBI	TiO_2_	2	0.081	175	---	[137]
PBI	Fe_2_TiO_5_	4	0.078	180	D	[138]
PBI	SiWA-SiO_2_	50	0.001	160	---	[141]
PBI	SiWA-SiO_2_	50	0.002	160	D	[141]
PBI	ZrP	15	0.096	200	D	[142]
PBI	BPO_4_	25	0.027	180	D	[142]
PBI	CsPOMo	30	0.120	160	D	[143]
PBI	CsPOW	30	0.100	160	D	[143]
PBI	CsSiOW	30	0.057	160	D	[143]
PBI	CsSiMo	30	0.051	160	D	[143]
PBI	BaZrO_3_	4	0.125	180	D	[144]

**Table 8:** Activation energy values for PBI, PBI@M[COSANE], and PBI@M[TPB] membranes.

Membrane	E_a_ (kJ/mol) T ∈ [20–100 °C]	E_a_ (kJ/mol)T ∈ [100–150 °C]
**PBI**@H[COSANE]	24.7 ± 0.7	5.6 ± 0.1
**PBI**@Li[COSANE]	19.1 ± 0.8	3.9 ± 0.4
**PBI**@Na[COSANE]	26.1 ± 1.2	5.2 ± 0.3
**PBI**@Li[TPB]	29.5 ± 0.9	6.1 ± 0.6
**PBI**@Na[TPB]	29.1 ± 1.6	7.3 ± 0.3
**PBI**	20.5 ± 2.3	----

**Table 9:** Composite membranes of polybenzimidazole derivates with GO derivates. MWCNT, multiwalled carbon nanotube. GO, graphene oxide; PGO, phosphonated GO; SGO, sulfonated GO; TrGO, GO bearing triazole groups.

Polymer	Filler	wt.%	σ_DC_ (S/cm)	T (°C)	PA	Ref
PBI	GO	2	0.170	165	---	[38]
PBI	TrGO	1.2	0.135	180	D	[159]
PBI	SGO	1	0.023	170	D	[126]
PBI-Py	PGO	1.5	0.076	140	D	[118]
PBI	CNT		0.074	180	D	[172]
PBI	MWCNT	0.2	0.08	160	D	[175]

**Table 10:** Composite membranes of polybenzimidazole with ionic liquids (ILs). HMI-Tf, 1-hexyl-3-methylimidazolium trifluoromethanesulfonate; BMIM, 1-butyl-3-methylimidazolium; SPAN, sulfonated polyaniline.

Polymer	ILs	wt.%	σ_DC_ (S/cm)	T (°C)	Ref
*o*-F_6_-PBI	HMI-Tf	3	0.016	250	[225]
PBI	[h-mim] NTf_2_	---	0.00186	190	[226]
PBI	BMIM	5	0.098	120	[227]
PBI	Cl	5	1.0 × 10^−4^	160	[227]
PBI	BF_4_	5	3 × 10^−6^	160	[227]
PBI	NCS	5	4 × 10^−7^	160	[227]
PBI	NTf_2_	5	6.5 × 10^−4^	160	[227]
PBI	[dema][TfO]_33_	33	<10^−4^	160	[229]
PBI	[dema][TfO]_50_	50	<10^−4^	160	[229]
OPBI	perovskite (SrCeO_3_)-PA	8	0.105	180	[230]
PBI-TGIC (5%)	SPAN	50	0.13	180	[145]
PBI-TGIC (10%)	SPAN	50	0.12	180	[145]

**Table 11:** Nanofibers polybenzimidazole derivates.

Polymer	σ_DC_ (S/cm)	T (°C)	PA	Ref
PBI	0.123	200	D	[240]
SO_2_-OPBI	0.067	160	D	[239]
m-PBI-PBz	0.170	160	D	[238]
PBI-basic SO_2_	0.003	200	---	[70]
